# CSNK2B modulates IRF1 binding to functional DNA elements and promotes basal and agonist-induced antiviral signaling

**DOI:** 10.1093/nar/gkad298

**Published:** 2023-04-24

**Authors:** Moe Matsumoto, Jennifer L Modliszewski, Kotomi Shinozaki, Reona Maezawa, Vincent M Perez, Yuki Ishikawa, Ryosuke Suzuki, Kevin L McKnight, Takahiro Masaki, Asuka Hirai-Yuki, Michinori Kohara, Stanley M Lemon, Sara R Selitsky, Daisuke Yamane

**Affiliations:** Department of Diseases and Infection, Tokyo Metropolitan Institute of Medical Science, Setagaya-ku, Tokyo 156-8506, Japan; QuantBio, LLC, Durham, NC 27705, USA; Department of Diseases and Infection, Tokyo Metropolitan Institute of Medical Science, Setagaya-ku, Tokyo 156-8506, Japan; Department of Diseases and Infection, Tokyo Metropolitan Institute of Medical Science, Setagaya-ku, Tokyo 156-8506, Japan; QuantBio, LLC, Durham, NC 27705, USA; Department of Diseases and Infection, Tokyo Metropolitan Institute of Medical Science, Setagaya-ku, Tokyo 156-8506, Japan; Department of Virology II, National Institute of Infectious Diseases, 162-8640 Tokyo, Japan; Lineberger Comprehensive Cancer Center, and Departments of Medicine and Microbiology & Immunology, The University of North Carolina at Chapel Hill, Chapel Hill, NC 27599-7292, USA; Department of Laboratory Medicine, The Jikei University School of Medicine, Tokyo 105-8461, Japan; Management Department of Biosafety, Laboratory Animal and Pathogen Bank, National Institute of Infectious Diseases, 162-8640 Tokyo, Japan; Department of Diseases and Infection, Tokyo Metropolitan Institute of Medical Science, Setagaya-ku, Tokyo 156-8506, Japan; Lineberger Comprehensive Cancer Center, and Departments of Medicine and Microbiology & Immunology, The University of North Carolina at Chapel Hill, Chapel Hill, NC 27599-7292, USA; QuantBio, LLC, Durham, NC 27705, USA; Department of Diseases and Infection, Tokyo Metropolitan Institute of Medical Science, Setagaya-ku, Tokyo 156-8506, Japan

## Abstract

Interferon regulatory factor 1 (IRF1) is a critical component of cell-intrinsic innate immunity that regulates both constitutive and induced antiviral defenses. Due to its short half-life, IRF1 function is generally considered to be regulated by its synthesis. However, how IRF1 activity is controlled post-translationally has remained poorly characterized. Here, we employed a proteomics approach to identify proteins interacting with IRF1, and found that CSNK2B, a regulatory subunit of casein kinase 2, interacts directly with IRF1 and constitutively modulates its transcriptional activity. Genome-wide CUT&RUN analysis of IRF1 binding loci revealed that CSNK2B acts generally to enhance the binding of IRF1 to chromatin, thereby enhancing transcription of key antiviral genes, such as *PLAAT4* (also known as *RARRES3*/*RIG1*/*TIG3*). On the other hand, depleting CSNK2B triggered abnormal accumulation of IRF1 at *AFAP1* loci, thereby down-regulating transcription of *AFAP1*, revealing contrary effects of CSNK2B on IRF1 binding at different loci. *AFAP1* encodes an actin crosslinking factor that mediates Src activation. Importantly, CSNK2B was also found to mediate phosphorylation-dependent activation of AFAP1-Src signaling and exert suppressive effects against flaviviruses, including dengue virus. These findings reveal a previously unappreciated mode of IRF1 regulation and identify important effector genes mediating multiple cellular functions governed by CSNK2B and IRF1.

## Graphical Abstract

**Graphical Abstract:**
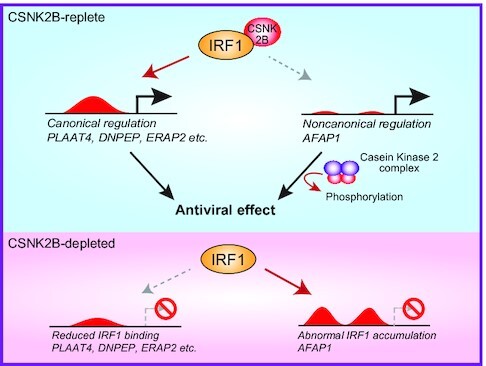
CSNK2B interacts directly with IRF1 and modulates the binding of IRF1 to chromatin, thereby promoting expression of antiviral genes.

## INTRODUCTION

Host inducible immune responses triggered by pathogen recognition via pattern recognition receptors (PRRs) comprise a key aspect of the innate immune system. By activating transcription of numerous antiviral effector genes, such responses mediate early protection against viral infections prior to the onset of adaptive immunity. By contrast, accumulating evidence supports the functional importance of constitutive innate immune defense as a mechanism of protection, acting even prior to the occurrence of host innate immune responses (1,2). Such constitutive mechanism, predominantly governed by interferon regulatory factor 1 (IRF1), provides more immediate restriction of pathogenic RNA virus infections than induced responses triggered via the PRRs, such as RIG-I-like receptors and Toll-like receptors, that lead to IRF3/7-dependent induction of type I and type III interferons (IFNs) (3). Localized to the nucleus, IRF1 maintains the basal expression of numerous antiviral genes by binding to specific DNA promoter elements (3). Gene knockout studies have revealed IRF1 to be a potent restriction factor constitutively expressed in hepatocytes. Compared with genetic depletion of IRF3 or IFN signaling components such as MAVS, IRF1 knockout caused more dramatic increases in permissiveness for infection by hepatotropic RNA viruses such as hepatitis A virus (HAV), hepatitis C virus (HCV), and dengue virus (DENV) (3). Importantly, the constitutive antiviral activity of IRF1 remained intact in cells lacking expression of MAVS, IRF3, and STAT1, demonstrating complete independence from the virus-induced responses (3). In contrast to IRF3 that is abundantly expressed at the basal level and undergoes post-translational modifications (PTMs) involving phosphorylation events upon pathogen recognition and activation of innate immune signaling, conflicting data have been reported as to whether and how phosphorylation modifies the activity and turnover of IRF1 (4–7). Due to its short half-life (20–40 min), the activity of IRF1 has been considered to be regulated primarily at the level of gene transcription and protein synthesis (8). The extent to which post-translational mechanisms control the function of IRF1 remains to be determined.

Here, we describe IRF1 interactome screens followed by genetic depletion analysis to identify host factors that directly interact with IRF1 and affect its ability to drive gene transcription. This led to the identification of CSNK2B, a regulatory subunit of casein kinase 2 (CK2), as an important host factor modulating IRF1 binding to a DNA promoter element driving efficient transcription of *PLAAT4* (a.k.a. *RARRES3*/*RIG1*/*TIG3*), a potent HAV restriction factor (1,3), thereby revealing that IRF1 function is regulated post-translationally by protein-protein interactions. Subsequent genome-wide analysis of CSNK2B-dependent IRF1 binding sites using Cleavage Under Targets & Release Using Nuclease (CUT&RUN) profiling supports a key regulatory role for CSNK2B in facilitating IRF1 binding to regulatory DNA elements controlling numerous genes related to innate immune defense responses. This comprehensive approach identified multiple cellular effectors and signaling pathways regulated by CSNK2B in both an IRF1- and CK2-dependent manner.

## MATERIALS AND METHODS

### Cells

PH5CH8 immortalized human primary hepatocytes, A549 human lung carcinoma cells, 293T and 293FT human embryonic kidney cells, and HepG2 and Huh-7.5 human hepatoma cells were mycoplasma-free and cultured in Dulbecco's modified Eagle's medium (DMEM), High Glucose supplemented with 10% fetal bovine serum (FBS), 1 × GlutaMAX-I and 1 × MEM Non-Essential Amino Acids Solution (Thermo Fisher Scientific) at 37°C in a 5% CO_2_ atmosphere. AML12 immortalized mouse hepatocytes were grown in DMEM/F-12 medium (Thermo Fisher Scientific) supplemented with 10% FBS, 1 × Insulin-Transferrin-Selenium (Gibco), 40 ng/ml dexamethasone and 2 mM GlutaMAX-I. Primary human hepatocytes (PXB cells) were purchased from PhoenixBio Co. and maintained in Leibovitz's L-15 medium (Wako) supplemented with 26 mM NaHCO_3_, 100 IU/ml insulin, 1 μM hydrocortisone and 10% FBS (Gibco).

### Reagents and antibodies

Primary antibodies to IRF1 (1:500 dilution, #8478), Src (1:500 dilution, #2123), and phospho-Src (Y416) (1:500 dilution, #6943) were from Cell Signaling Technology; DYKDDDDK (Clone 1E6, 1:1000 dilution, 018–22381) and GAPDH were from Wako (Clone 5A12; 1:5000 dilution, 016-25523); CSNK2B (1:2000 dilution, A301-984A) and CSNK2A2 (1:2000 dilution, A300-199A) were from Bethyl Laboratories; and CSNK2A1 (1:2000 dilution, 10992-1-AP) and AFAP1 (1:500 dilution, 14544-1-AP) were from Proteintech. IRDye 680 or 800 secondary antibodies including #926-32211, #926-32212, #926-32214, #926-68020 and #926-68073 (1:20 000 dilution) were from LI-COR.

CX-4945 (Silmitasertib) was purchased from Selleck. Puromycin and Hygromycin B Gold were from InvivoGen. Pyridone 6 (JAK Inhibitor I) was from Cayman Chemical. All-trans-retinoic acid was from Wako. Recombinant human IFN-γ and CSNK2B were from Pepro Tech and NKMAX, respectively. PSI-7977 (Sofosbuvir) was from ChemScene. Lambda protein phosphatase was obtained from Bio Academia. miR-122 mimics were synthesized by Dharmacon and transfected by electroporation as miRNA/miRNA* duplexes as described (9). Cell viability was determined using Cell Counting Kit-8 (DOJINDO, Japan) on 96-well plates according to the manufacturer's protocol.

### Viruses

A cell culture-adapted variant of HM175 strain HAV (HM175/18f) and the HAV/NLuc reporter virus, 18f/NLuc were prepared as described before (3). A chimeric HAV reporter virus, p16-18fSP/NLuc, containing the 2B and partial 2C sequence derived from 18f/NLuc in the p16 background, was constructed using the SacI and PflmI sites. pHAV-Luc and pHAV-LucΔ3D were described previously (3). The secretory NLuc-coding sequence followed by the foot-and-mouth disease virus 2A protease–coding sequence was inserted between p7 and NS2 in pJFH1-QL to generate pJFH1-QL/NLuc as described (10). Dengue virus serotype 2 (o1Sa-054 strain) and Zika virus (MR-766 strain) were propagated in Huh-7.5 or Vero cells, respectively, as described (3).

DENV/NLuc reporter virus carrying a subgenomic RNA containing NS1-5 region fused with a NLuc reporter was produced by co-transfecting with plasmids expressing capsid and prME as described (11).

### Other plasmids

The lentiviral transfer plasmids encoding AFAP1 were created by PCR amplifying the host genes using cDNA derived from PH5CH8 cell total RNA as template and primers flanked by XbaI and NheI restriction sites, and ligated into pCSII-EF-MCS vector. The NLuc reporter vector pNL-4 × IRF1 was prepared by annealing oligonucleotides containing IRF1 binding motifs derived from the *PLAAT4* promoter followed by a minimal TATA promoter, 5′-CTAGCAAAAGGAAAGTGAAAGTGAAATTCAAAAGGAAAGTGAAAGTGAAATTAAGCTTAGAGGGTATATAATGGAAGCTCGACTTCCAG-3′ and 5′-AGCTCTGGAAGTCGAGCTTCCATTATATACCCTCTAAGCTTAATTTCACTTTCACTTTCCTTTTGAATTTCACTTTCACTTTCCTTTTG-3′, and inserted into pNL2.3 plasmid (Promega) using NheI and HindIII restriction sites.

### Viral RNA transcription and transfection

In vitro transcription of HAV or HCV RNA was carried out using T7 RiboMAX™ Express Large Scale RNA Production System (Promega) as per manufacturer's protocol. Transfection of viral RNA (5 μg) was performed in a Gene Pulser Xcell Total System (Bio-Rad) or using TransIT-mRNA Transfection Kit (Mirus) for subgenomic HAV-Luc RNA as described (3,12).

### Lentivirus production and transduction

Lentiviral transfer vector was co-transfected with standard packaging plasmids into 293T cells using PEI MAX reagent (Polysciences) and the supernatant fluids harvested at 72 h were filtered through a 0.22 μm syringe filter. Production of sgRNA CRISPR/Cas9 lentivirus was similarly carried out by co-transfecting 293T cells with sgRNA expressing vectors listed in Supplementary Table S4. Lentivirus transduction was performed by supplementation of 8 μg/ml polybrene, followed by antibiotic selection with 6 μg/ml puromycin. Antibiotic-resistant bulk cell populations were used for experiments to avoid clonal biases.

### RNA extraction and quantitative RT-PCR

Total RNA extraction was performed with the RNeasy mini Kit (Qiagen). Detection of HAV genome RNA was carried out by a two-step quantitative RT-PCR analysis with the ReverTra Ace qPCR RT Kit and THUNDERBIRD Next SYBR qPCR Mix (TOYOBO) using specific primers 5′-GGTAGGCTACGGGTGAAAC-3′ and 5′-AACAACTCACCAATATCCGC-3′. Quantification of cellular genes was performed with the primer pairs listed in Supplementary Table S5. DENV and ZIKV RNA levels were quantified using specific primer pairs targeting DENV genome RNA, 5′-ACACCACAGAGTTCCATTACAGA-3′ and 5′-CATCTCATTAAAGTCGAGGCC-3′, or ZIKV genome RNA, 5′-AARTACACATACCARAACAAAGTGGT-3′ and 5′-TCCRCTCCCYCTYTGGTCTTG-3′ respectively, using Luna Universal One-Step RT-qPCR Kit (NEB).

### Immunoblots

Western blotting was performed with standard methods. Odyssey CLx Infrared Imaging System (LI-COR Biosciences) was used for visualization. Lysates for Phos-tag gel analysis was prepared in Tris-buffered saline containing 1% Nonidet *P*-40 supplemented with EDTA-free Complete protease inhibitor cocktail (Roche) and resolved on a 10% Phos-tag SDS-PAGE gel (Wako, #190-16721).

### RNA interference

siRNA pools listed in Supplementary Table S3 were obtained from Dharmacon, Thermo Fisher Scientific, or Sigma-Aldrich and transfected into cells using Lipofectamine RNAiMAX Transfection Reagent (Thermo Fisher Scientific) at a final concentration of 20 nM according to the manufacturer's protocol.

### Luciferase assay

NLuc or Firefly luciferase (FLuc) activity was measured using Nano-Glo Luciferase Assay System or Luciferase Assay System (Promega), respectively, as per the manufacture's protocol. Luminescence was analyzed on a Mithras LB940 (Berthold).

### Purification of recombinant IRF1 expressed in mammalian cells

FLAG-tagged IRF1 proteins were ectopically expressed in 293FT cells grown on 15 cm dishes using PEI MAX reagent (Polysciences). Cells transfected with empty vector were processed in parallel. Twenty-four hours after transfection, the cells were scraped into a lysis buffer (50 mM Tris–HCl, pH 7.5, 150 mM NaCl, 1 mM EDTA, 50 mM sodium fluoride, 1 mM Na_3_VO_4_ and 1% Triton X-100) supplemented with a Complete protease inhibitor cocktail (Roche). Clarified lysates were subsequently purified by binding to Anti-FLAG M2 affinity agarose gel (Sigma), followed by elution with the FLAG peptide (0.2 mg/ml). The resulting eluate was concentrated using an Amicon Ultra 10K Centrifugal filters (Millipore) and diluted in wash buffer (Tris-buffered saline containing 1% Nonidet *P*-40). This procedure was repeated five times to reduce the concentration of the FLAG peptide.

### Proteomics analysis

Affinity- purified samples (15 μl) were treated with 1 μl 50 mM tris-(2-carboxyethyl) phosphine and incubated at 60°C for 2 h, followed by addition of 0.5 ml 200 mM Methylmethanethiosulfonate and incubated at RT for 15 min. Protein samples at pH 7.5–8.5 were then digested at 37°C for 2 h with Lysyl Endopeptidase, Mass Spectrometry Grade (Lys-C, 200 ng μl^−1^, Wako) as per manufacturer's instruction, and treated with 100 μl 2% acetonitrile/0.1% trifluoroacetic acid (TFA) in water. Samples concentrated by SpeedVac were used for LC-MS/MS analysis. To identify phosphopeptides derived from IRF1, gel lanes were sliced in 2-mm intervals, and slices were cubed and destained with 50 mM ammonium bicarbonate/30% acetonitrile. Gels were dehydrated in 50 mM ammonium bicarbonate/40% acetonitrile and dried by Speed Vac. After dried gels were immersed on ice in 12.5 ng/ml trypsin (Promega) in 50 mM ammonium bicarbonate, proteins were in-gel digested overnight at 37°C. Peptides were extracted with 70% acetonitrile/0.1% TFA, and concentrated by SpeedVac.

Analytical samples were loaded onto a 75 μm Acclaim PepMap (ThermoFisher Scientific) packed with 3 cm of a C18 analytical column (3 μm particles, NTCC-360, Nikkyo Technos). Peptides were gradient-eluted using 0.1 M formic acid/80% (vol/vol) acetonitrile in water at a flow rate of 300 nL/min into an TripleTOF 5600+ (AB Sciex Instruments). Resulting data were analyzed using ProteinPilot Software 5.0.1 (Sciex, USA) and searched against a database ‘uniprot_sprot_can + iso_20100622’, as well as a decoy database of reversed protein sequences.

NetPhos server 3.1 was used to analyze candidate CK2-phosphorylation sites in IRF1 (13)

### Oligonucleotide pull-down analysis

PH5CH8 cells transfected with either *CSNK2B* or control siRNAs were lysed in HKMG buffer (10 mM HEPES pH 7.9, 100 mM KCl, 5 mM MgCl_2_, 10% glycerol, 1 mM DTT and 1% Nonidet *P*-40) 3 day post-transfection. Lysates were incubated at 4°C for 2 h with 5′-biotinylated double-stranded oligonucleotides spanning the 3 tandem repeats of IRF1 binding sequence derived from the *PLAAT4* promoter, 5′-CTAGCAAAAGGAAAGTGAAAGTGAAATTCAAAAGGAAAGTGAAAGTGAAATTCAAAAGGAAAGTGAAAGTGAAATT-3′ (1 μg), in the presence of 1 μg poly(dI–dC) (Thermo Fisher Scientific), followed by addition of 25 μl Magnosphere MS160/Streptavidin (JSR Life Science). Another 1 h later, the beads were washed with HKMG buffer five times, followed by Western blotting analysis of the DNA probe-bound IRF1 protein.

### CUT&RUN assay

CUT&RUN was performed on 5 × 10^5^ PH5CH8 cells transfected with *CSNK2B* versus non-target control siRNAs using CUT&RUN Assay Kit (Cell Signaling Technology, #86652) as per manufacturer's protocol. In brief, trypsinized cells were bound to Concanavalin A beads, permeabilized with digitonin, and incubated with IRF1 antibody (Cell Signaling Technology, #8478, 1:25 dilution) overnight at 4°C on a thermo shaker. On the next day, Protein A-fused micrococcal nuclease was incubated for 30 min at 4°C to bind IRF1 antibody and digest bound sites, followed by incubation at 37°C for 10 min to release digested DNA fragments. DNA was purified using NucleoSpin Gel and PCR Clean-up (MACHEREY-NAGEL) and eluted in 50 μl nuclease-free water.

### ChIP-seq and bioinformatics analysis

Integrity of DNA samples was determined with a TapeStation HS D5000 Screen Tape (Agilent). Library for next-generation sequencing was prepared using TruSeq ChIP Library kit. Paired-end 2 × 150 bp sequencing was performed on an NovaSeq6000 platform at Macrogen Japan (Kyoto, Japan). Sequencing quality were comparable for all samples. Raw paired-end reads were trimmed of poor quality bases and adaptors via fastp (v0.23.2) (14) with adaptor detection for paired-end reads and otherwise default parameters. Trimmed reads were aligned to Hg38 via bowtie2 (v 2.2.9) (15) with settings of very sensitive local and a maximum fragment length of 800.

Differential peak abundance was conducted in R statistical programming environment. Global peaks were identified via csaw (v1.28.0) (16). Briefly, reads were first counted into windows with window width set to 10, and fragment length set to 160. Optimal fragment length was determined via csaw ‘correlatedReads’ output. To define peaks, reads were again counted into 10 kb bins and original peaks falling below a global log_2_ fold change of 5 were excluded. Read counts for this final set of peaks were normalized for library size via trimmed mean of *M*-values (TMM) in csaw. Differential peak abundance was tested via a quasi-likelihood F-test implemented in edgeR (17) (v.3.36.0). Corrections for multiple comparisons are performed by merging regional peaks with a maximal adjacent distance of 100 bp and a maximal overall width of 5 kb. Peaks were annotated with ChIPseeker (v1.30.3) (18). Motif enrichment analysis was performed with Homer (v4.10) (19), using the ‘findMotifsGenome.pl’ function. Homer was run separately for all nominally significant peaks that were high in *CSNK2B* siRNA and high in control siRNA with default parameters.

Gene ontology enrichment analysis was performed using DAVID (The Database for Annotation, Visualization and Integrated Discovery) 6.8.

### Statistical analysis

Unless noted otherwise, the error bars represent the standard deviation and are shown for experiments with *n*  =  3 or greater. All between-group comparisons were carried out by ANOVA or two-tailed *t*-test using Prism 6.0 software (GraphPad Software, Inc.). The *P*-values were calculated from 3 biological replicates.

## RESULTS

### Purification of IRF1 protein expressed in mammalian cells

**Figure 1.**
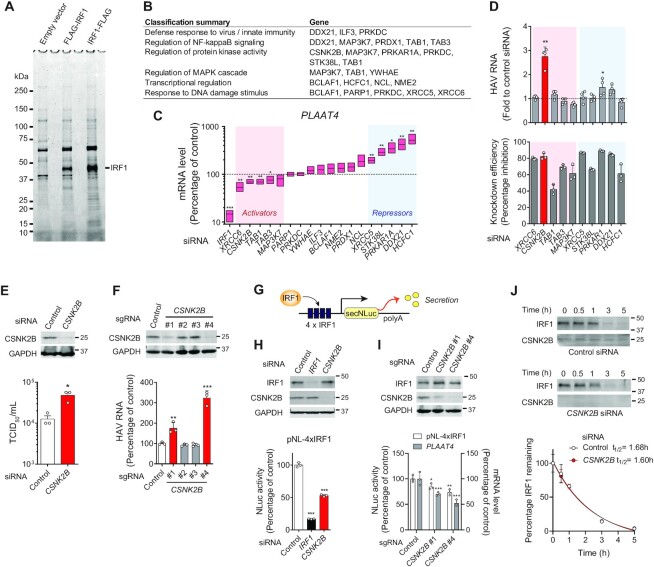
IRF1-associated cellular proteins identified by MS and functional validation of CSNK2B as an IRF1 cofactor. (**A**) Coomassie brilliant blue staining of affinity-purified FLAG-tagged IRF1 proteins on SDS-PAGE gels. The gel was imaged in the 700 nm channel on the Odyssey Infrared Imaging System. (**B**) Gene ontology analysis and functional annotation of IRF1-associated proteins identified by MS. (**C**) Validation of cellular proteins affecting the endogenous *PLAAT4* transcript level by siRNA knockdown experiments. RNA levels were determined by RT-qPCR assays of RNA extracted from PH5CH8 cells transfected with siRNA pools targeting indicated genes for 72 h. *P < 0.05, **P < 0.01, ***P < 0.0001 versus control (line at mean, *n* = 3, two-tailed Student's *t*-test). (**D**) PH5CH8 cells were transfected with selected siRNA pools and infected 48 h later with hepatitis A virus (HAV) at an m.o.i. of 10. Relative abundance of HAV RNA was determined 4 day post-infection (d.p.i.) by RT-qPCR (upper panel, *n* = 4). Knockdown efficiency of target genes was determined 6 days post-siRNA transfection (lower panel, *n* = 3). *P < 0.05, **P < 0.01 versus control (one-way ANOVA with Dunnett's multiple comparisons test). (**E**) HAV/NLuc (18f/NLuc) infectious titers released from PH5CH8 cells transfected with CSNK2B siRNA or control as in (D) were determined 5 d.p.i. *P < 0.05 (*n* = 3, two-tailed Student's *t*-test). (**F**) HAV RNA levels at 4 d.p.i. in PH5CH8 cells expressing *CSNK2B* versus control sgRNAs. **P < 0.01, ***P < 0.0001 versus control (*n* = 3, one-way ANOVA with Dunnett's multiple comparisons test). (**G**) Schematic representation of secretory Nanoluciferase (NLuc) reporter analysis of 4 × IRF1-NLuc stably transfected in PH5CH8 cells. (**H**) NLuc activities in cells transfected with *IRF1*, *CSNK2B* and control siRNAs. The promoter activity was determined 3 days post-transfection. ***P < 0.0001 versus control (*n* = 3, one-way ANOVA with Dunnett's multiple comparisons test). **(I)** NLuc activities and *PLAAT4* mRNA levels in PH5CH8 cells expressing *CSNK2B* versus control sgRNAs. The promoter activity was determined 3 days post-transduction. *P < 0.05, **P < 0.01, ***P < 0.0001 versus control (one-way ANOVA with Dunnett's multiple comparisons test). (**J**) Stability of IRF1 protein in PCH5CH8 cells transfected with CSNK2B siRNA or control after treatment with 50 μg/ml puromycin. Data were fit to a one-phase decay model (*n* = 3, *R*^2^ = 0.9588–0.9832).

To identify cellular factors affecting the constitutive antiviral activity of IRF1, we expressed IRF1 tagged with an N-terminal (FLAG-IRF1) or C-terminal (IRF1-FLAG) FLAG epitope in 293FT cells. We confirmed that ectopically expressed FLAG-tagged IRF1 proteins were transcriptionally active and capable of driving expression of its primary target gene, *PLAAT4*, in transfected 293FT cells (Supplementary Figure S1A) (3), and thus suited for isolating cellular factors affecting IRF1 activity to drive gene expression. Affinity purification of FLAG-tagged IRF1 by means of anti-FLAG M2 antibody agarose beads, followed by elution using FLAG peptides and Coomassie Brilliant Blue staining of gels showed numerous bands co-purified with the FLAG-tagged IRF1 (Figure 1A).

### Proteomics identification of candidate IRF1-interacting proteins

The proteins co-precipitated with the IRF1 proteins were analyzed by liquid chromatography–mass spectrometry (LC/MS). Analysis of a control sample processed via identical procedures from empty vector-transfected lysates identified several proteins nonspecifically bound to beads and eluted by FLAG peptides (Supplementary Table S1A). By subtracting these as background, we narrowed down the list of host proteins specifically associated with IRF1 (Supplementary Table S1B, C). These candidate IRF1-specific interactors represented multiple, diverse functional groups, including proteins involved in the cellular response to DNA damage stimulus, defense response to virus, the proteasome, ribonucleoproteins, spliceosome, cytoskeleton, transcriptional regulation and regulation of mitogen-activated protein kinase (MAPK) and NFκB signaling (Supplementary Figure S1B). Of these, we considered factors related to the spliceosome and ribonucleoproteins likely to be associated with DNA bound to IRF1, rather than IRF1 itself, and proteasome components likely involved in the degradation of short-lived IRF1 (*t*_1/2_ = 50 min in HEK293 cells) (6). Thus, we prioritized proteins linked to host defense responses, innate immune signaling and transcriptional regulation for validation experiments by RNA interference (Figure 1B).

### Validation of host factors mediating the antiviral activity of IRF1

We used siRNA pools targeting the genes listed in Figure 1B to deplete their expression in T antigen-transformed adult human hepatocytes (PH5CH8 cells) and assessed the impacts on the transcriptional activity of endogenous IRF1. An siRNA pool targeting *IRF1* itself was used as a positive control for inhibition of IRF1 in these functional experiments. Three days post-siRNA transfection, total RNA was extracted and analyzed for mRNA levels of *PLAAT4*, the gene most down-regulated upon IRF1 depletion (3), as an endogenous indicator reflecting IRF1 activity. Whereas about half of the genes targeted by the siRNAs were functionally neutral, silencing *XRCC6*, *CSNK2B*, *TAB1* and *TAB3* significantly reduced *PLAAT4* transcription, suggesting an IRF1 transcriptional activator function for these genes (Figure 1C). Conversely, enhanced *PLAAT4* expression was observed in cells depleted of *XRCC5*, *STK38L*, *PRKAR1A*, *DDX21* and *HCFC1*. Thus, these genes were categorized as potential IRF1 repressors (Figure 1C).

To assess the functional roles of these candidate IRF1 regulators in control of viral replication, siRNA-transfected PH5CH8 cells were challenged with HAV, a virus highly sensitive to IRF1-dependent antiviral signaling (3). Intracellular viral RNA levels were measured 4 days later by reverse-transcription quantitative PCR (RT-qPCR). Consistent with a previous finding that HAV replication in PH5CH8 cells is profoundly restricted by cell-intrinsic antiviral signaling driven by constitutively-expressed IRF1 (3), silencing the expression of candidate IRF1 repressors did not further reduce HAV RNA levels (Figure 1D). Although silencing candidate IRF1 activators was expected to increase HAV replication, only depletion of *CSNK2B* resulted in significant increases in HAV RNA levels and enhanced production of infectious virus (Figure 1D, E). We further validated the functional importance of CSNK2B by using the CRISPR-Cas9 system to generate four independent *CSNK2B* knockout cell pools. Two out of four single-guide RNAs (sgRNAs) successfully reduced the protein expression of CSNK2B, and this decrease was paralleled by an increase in HAV RNA levels (Figure 1F). Collectively, these results reveal CSNK2B to be an anti-HAV factor that enhances *PLAAT4* transcription.

To confirm that CSNK2B regulates *PLAAT4* via IRF1, we engineered an IRF1 reporter PH5CH8 cell line in which four tandem repeats of an IRF1-responsive element derived from the *PLAAT4* promoter drives expression of secreted Nanoluciferase (NLuc) in response to IRF1 binding (Figure 1G). IRF1-dependent expression of the NLuc reporter was well validated by depletion of *IRF1* (Figure 1H). Importantly, siRNA-mediated *CSNK2B*-depletion inhibited the NLuc reporter ∼50% (Figure 1H). The decrease in the NLuc reporter, as well as *PLAAT4* transcript levels, also correlated with the reduced expression of CSNK2B protein in CRISPR-Cas9 knockout cell pools without affecting the IRF1 abundance (Figure 1I). Indeed, CSNK2B depletion did not affect the stability of IRF1 protein in PH5CH8 cells: the half-life of IRF1 protein in cells transfected with control siRNA was 1.6 h versus 1.68 h for cells transfected with *CSNK2B* siRNA (Figure 1J). These results suggest that silencing CSNK2B expression suppresses IRF1 activity post-translationally, thereby down-regulating *PLAAT4* expression.

### CSNK2B interacts with IRF1 but does not regulate IRF1 phosphorylation status

**Figure 2.**
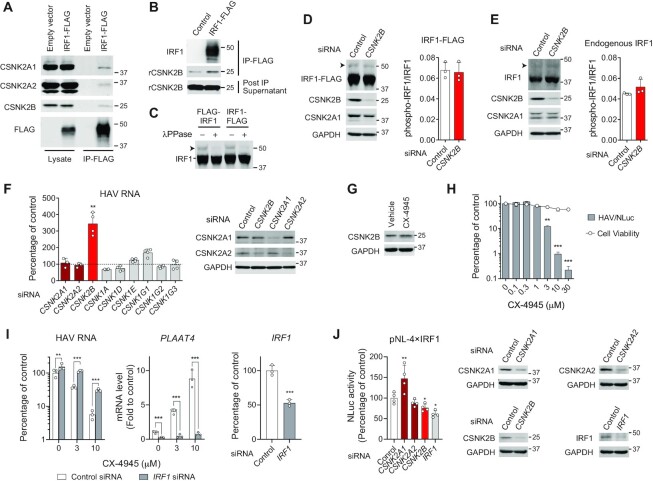
CSNK2B interacts with IRF1 and regulates its antiviral activity independently of phosphorylation. (**A**) 293FT cell lysates expressing IRF1-FLAG or empty vector was immunoprecipitated with anti-FLAG M2 antibody. Proteins eluted from the precipitates were subjected to an SDS-PAGE followed by western blotting with specific antibodies against CK2 components. (**B**) Pull-down analysis showing direct interaction between purified IRF1-FLAG and recombinant human CSNK2B (rCSNK2B) proteins. (**C**) Phos-tag SDS-PAGE of FLAG-tagged IRF1. PH5CH8 cell lysates were subjected to immunoblotting before (−) and after (+) digestion with lambda protein phosphatase (λ PPase). An arrowhead shows phospho-IRF1. (**D**, **E**) Phos-tag gel analysis of ectopically expressed IRF1-FLAG in 293FT cells (**D**) or endogenously expressed IRF1 in PH5CH8 cells (**E**). Means ± S.D. of values for abundance of phospho-IRF1 relative to non-phosphorylated IRF1 are shown on right (*n* = 3). (**F**) PH5CH8 cells were transfected with siRNAs targeting CK components and infected 48 h later with HAV at an m.o.i. of 10. Percentage of HAV RNA levels relative to non-target control siRNA was determined 4 d.p.i. by RT-qPCR. Immunoblots showing depletion of catalytic CK2 subunits are shown on right. **P < 0.01 versus control (*n* = 3 or 4, one-way ANOVA with Dunnett's multiple comparisons test). (**G**) Immunoblots showing CSNK2B protein abundance in Huh-7.5 cells treated with 10 μM CX-4945 for 24 h. (**H**) Effects of CX-4945 on replication of HAV/NLuc (18f/NLuc) in Huh-7.5 cells and cell viability. **P < 0.01, ***P < 0.0001 versus control (*n* = 3, one-way ANOVA with Bonferroni's multiple comparisons test). (**I**) Effects of CX-4945 on HAV replication and *PLAAT4* expression in *IRF1*-depleted and control Huh-7.5 cells. **P < 0.01, ***P < 0.0001 (*n* = 3, two-way ANOVA with Sidak's multiple comparisons test). (**J**) NLuc reporter analysis of Huh-7.5 cells expressing NLuc reporter (pNL-4×IRF1). Cells were transfected with indicated siRNAs for 48 h, and relative NLuc values secreted at 48–72 h post-transfection are shown. Immunoblots showing depletion of each siRNA target are shown on right. *P < 0.05, **P < 0.01 versus control (*n* = 4, one-way ANOVA with Dunnett's multiple comparisons test).

Given the impact of CSNK2B on IRF1 activity, we sought to determine whether it interacts with IRF1 protein. CSNK2B functions as the regulatory subunit of casein kinase 2 (CK2), a tetrameric complex composed of two catalytic α-subunits (CSNK2A1 and CSNK2A2) and two regulatory β-subunits (CSNK2B). We found IRF1-FLAG specifically co-immunoprecipitated with endogenous CSNK2B, as well as with the catalytic subunits, implying that CSNK2B might interact with IRF1 as part of a CK2 complex (Figure 2A). Importantly, an *in vitro* pull-down experiment using purified IRF1-FLAG and recombinant CSNK2B proteins indicated a direct interaction between the two proteins (Figure 2B). A previous study suggested CK2 might activate IRF1 by phosphorylating the C-terminal activation domain based on data from an *in vitro* kinase assay (5). However, unlike IRF3 that is heavily phosphorylated upon activation, phosphorylated IRF1 could not be detected as distinct bands by conventional Western blotting. Thus, we employed Phos-tag SDS-PAGE for sensitive detection of the phosphorylated form, and successfully detected a slowly migrating band of IRF1, which was sensitive to λ phosphatase treatment (Figure 2C). Quantifying the ratio of phosphorylated versus non-phosphorylated forms of ectopically expressed IRF1 in 293FT cells, as well as endogenous IRF1 in PH5CH8 cells showed that depleting expression of *CSNK2B* did not affect the IRF1 phosphorylation state (Figure 2D, E). Moreover, siRNA-mediated depletion of the catalytic subunits, *CSNK2A1* and *CSNK2A2*, as well as individual CK1 isoforms, did not affect HAV RNA levels in PH5CH8 cells (Figure 2F). Pharmacologic inhibition of the catalytic activity of CK2 using CX-4945 (Silmitasertib) in PH5CH8 cells reduced the abundance of CSNK2B protein and concomitantly inhibited IRF1-driven transcription of *PLAAT4*, thereby enhancing HAV replication in an IRF1-dependent fashion (Supplementary Figure S2A, B). While this unexpected reduction of CSNK2B by CX-4945 makes it difficult to dissect the role played by the catalytic CK2 subunits versus CSNK2B in control of IRF1 activity (Supplementary Figure S2A), CX-4945 treatment in human hepatoma (Huh-7.5) cells did not affect CSNK2B abundance and dose-dependently suppressed HAV replication (Figure 2G, H). Importantly, the anti-HAV effects of CX-4945 observed in Huh-7.5 cells require IRF1 and are associated with enhanced expression of IRF1-dependent *PLAAT4* (Figure 2I). siRNA depletion analysis showed that depletion of CSNK2A1 versus CSNK2B had contrasting effects on the IRF1 activity in Huh-7.5 cells: the former increased its activity whereas the latter decreased (Figure 2J).

Mass spectrometry analysis of affinity-purified IRF1 ectopically expressed in 293FT cells identified Ser87, Thr266, Ser273, Ser282 and Thr311 as phosphorylation sites (Supplementary Figure S3A). However, alanine substitutions at these sites did not affect the capacity of IRF1 to drive NLuc reporter gene expression in both PH5CH8 and 293T cell lines stably transfected with the pNL-4×IRF1 reporter construct (Supplementary Figure S3B). Similarly, alanine substitutions at six additional serine/threonine residues within IRF1 that are predicted to be targeted by CK2 did not impair IRF1-driven expression of NLuc (Supplementary Figure S3C, D). Together, these data indicate that phosphorylation at these sites is not essential for IRF1 transcriptional activity.

### CSNK2B restricts HAV genome replication via *PLAAT4*

**Figure 3.**
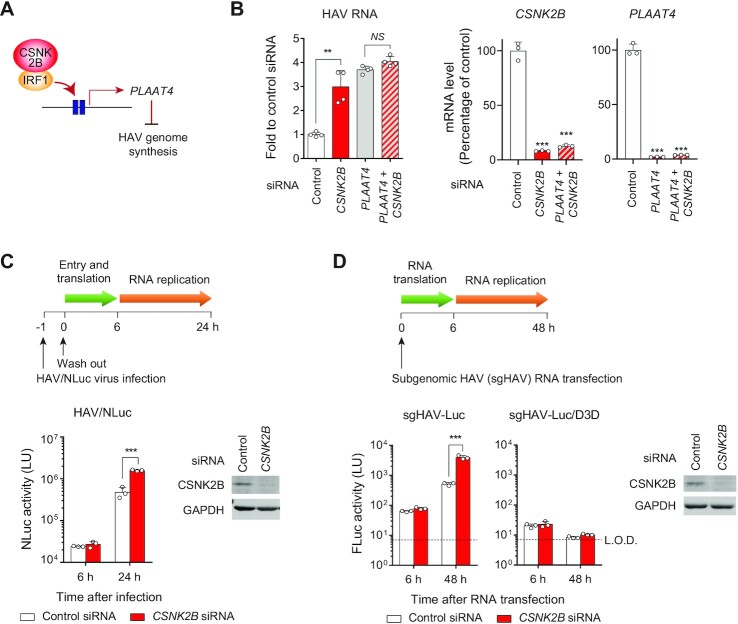
CSNK2B promotes IRF1-dependent expression of *PLAAT4* and restricts HAV RNA replication. (**A**) Scheme showing the CSNK2B/IRF1-mediated restriction of HAV replication via *PLAAT4*. (**B**) Relative HAV RNA levels at 5 d.p.i. in PH5CH8 cells transfected with siRNA targeting either *CSNK2B* or *PLAAT4* alone or the combination of 2 siRNAs (bottom). NS, not significant. **P < 0.01 (*n* = 4, two-tailed Student's *t*-test). Knockdown efficiency of *CSNK2B* and *PLAAT4* is shown on right. ***P < 0.0001 versus control (*n* = 3, one-way ANOVA with Dunnett's multiple comparisons test). (**C**) A549 cells transfected with *CSNK2B* versus control siRNAs were challenged with HAV/NLuc (18f/NLuc). NLuc activities at indicated time points post-infection are shown. Light units (LU) of noninfected lysates was 28.25 ± 9.83 (mean ± S.D.). Immunoblots showing depletion of CSNK2B are shown on right. ***P < 0.0001 (*n* = 3, two-way ANOVA with Sidak's multiple comparisons test). (**D**) Firefly luciferase (FLuc) activity produced from transfected subgenomic HAV-Luc (sgHAV-Luc) RNA or its replication-incompetent mutant (Δ3D) in Huh-7.5 cells depleted of *CSNK2B* versus control. Immunoblots showing depletion of CSNK2B are shown on right. L.O.D., limit of detection showing LU of non-transfected lysates. ***P < 0.0001 (*n* = 3, two-way ANOVA with Sidak's multiple comparisons test).

Our previous work demonstrated that *PLAAT4*, among other IRF1-regulated genes, is a dominant restriction factor that restricts HAV replication (3). Thus, we questioned whether the increase in IRF1-dependent *PLAAT4* expression is sufficient to explain the anti-HAV effect of CSNK2B (Figure 3A). *CSNK2B* depletion caused little increase in HAV RNA levels in the absence of *PLAAT4* (Figure 3B). By contrast, silencing the murine orthologue, *Csnk2b*, failed to increase HAV replication in the mouse hepatocyte cell line AML12, consistent with the absence of a *PLAAT4* orthologue in mice (Supplementary Figure S4). In agreement with the previous finding that PLAAT4 specifically restricts HAV RNA replication (3), we found that silencing *CSNK2B* expression relieves a block at a post-entry and post-translation step in replication of an NLuc-expressing HAV reporter virus (HAV/NLuc; Figure 3C), and that it enhanced replication of a subgenomic RNA replicon lacking capsid protein sequence (Figure 3D). Altogether, these results show that PLAAT4-dependent restriction accounts for the majority of the anti-HAV effects regulated by CSNK2B.

### CSNK2B promotes constitutive protection against HAV infection in primary human hepatocytes

**Figure 4.**
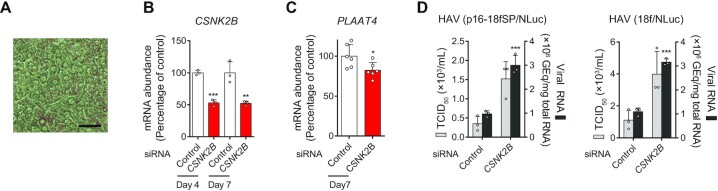
CSNK2B regulates *PLAAT4* expression and down-regulates HAV replication in primary human hepatocytes. (**A**) Phase contrast microscopy of primary human hepatocytes. Scale bar, 20 μm. (**B**) Effect of *CSNK2B* siRNA on *CSNK2B* mRNA levels at indicated time points after siRNA transfection. **P < 0.01, ***P < 0.0001 versus control (*n* = 3, two-tailed Student's *t*-test). (**C**) Effect of *CSNK2B* siRNA on *PLAAT4* mRNA levels at 7 days after transfection. *P < 0.05 versus control (*n* = 6, two-tailed Student's *t*-test). (**D**) Replication of HAV RNA (p16-18fSP and 18f strains that express NLuc) and infectious virus titers at 6 days p.i. in primary human hepatocytes transfected with indicated siRNAs. GEq, genome equivalents. ***P < 0.0001 versus control (*n* = 3, two-tailed Student's *t*-test).

We next attempted to validate the functional importance of CSNK2B on constitutive antiviral defense (20). Because the absence of the important antiviral effector, *PLAAT4*, in rodents precluded use of a mouse infection model, we examined the impact of silencing *CSNK2B* expression on HAV replication in primary human hepatocytes (PHHs) (Figure 4A). Experiments in PHHs were carried out in the continued presence of pyridone 6, a pan-JAK inhibitor, to diminish the induced IFN response and unmask the role of CSNK2B in constitutive defense. PHHs were transfected with siRNAs 1 day after plating, and sustained knockdown of *CSNK2B* was observed over the ensuing 7 days (Figure 4B). Despite only modest (50%) *CSNK2B* depletion, we observed reduced expression of *PLAAT4* (Figure 4C), significantly increased viral RNA levels, and enhanced production of infectious virus using both a low-passage noncytopathic (p16-18fSP/NLuc) and high-passage cytopathic (18f/NLuc) virus variants (Figure 4D). These results show that the CSNK2B-IRF1-PLAAT4 axis is constitutively active in restricting HAV infection in PHHs, arguing for the functional relevance of this restriction pathway in primary cells, the natural target for HAV.

### CSNK2B regulates both basal and agonist-induced activities of IRF1

**Figure 5.**
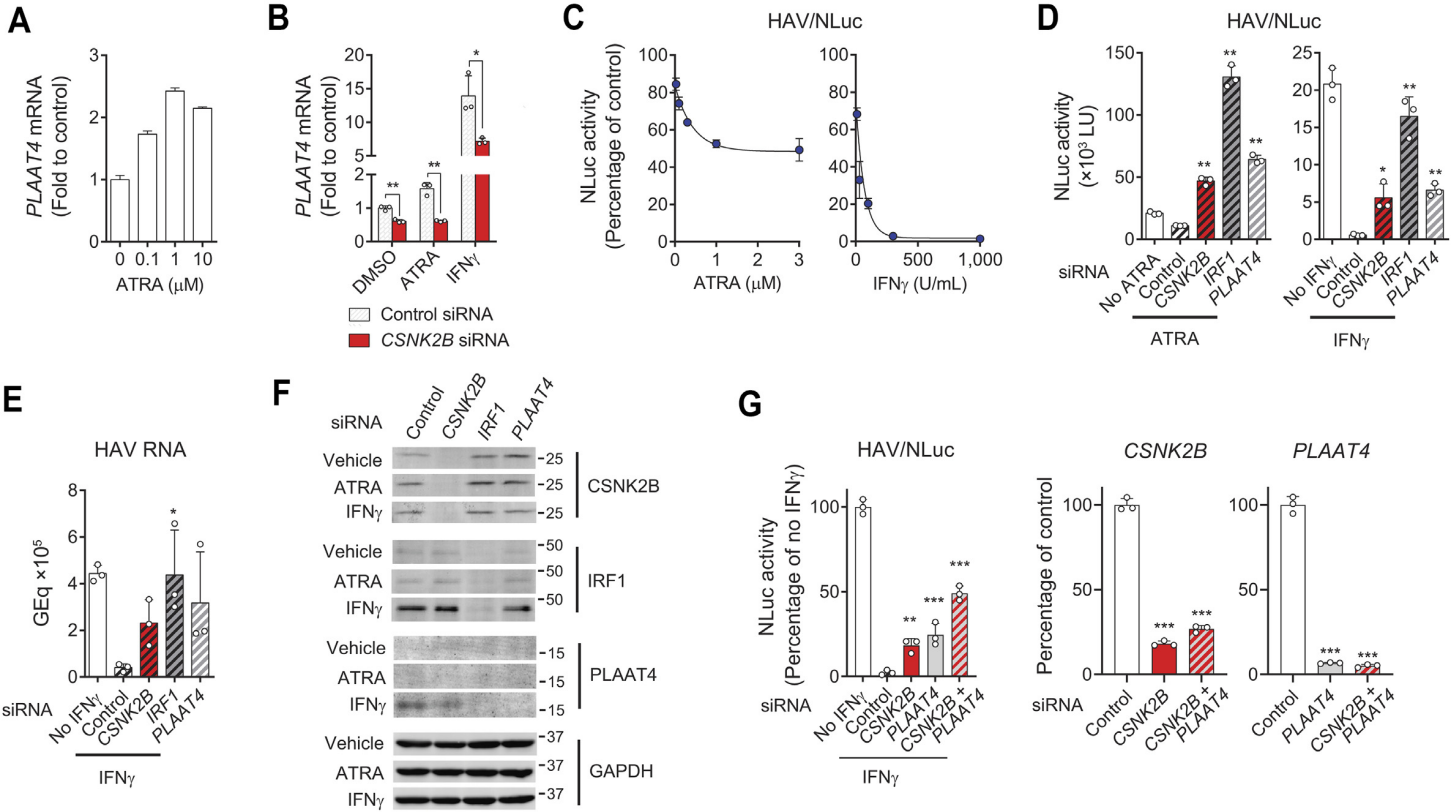
CSNK2B regulates agonist-induced suppression of HAV via *PLAAT4*. (**A**) RT-qPCR determination of *PLAAT4* mRNA levels in A549 cells treated with various doses of all-trans retinoic acid (ATRA) for 24 h (*n* = 2). (**B**) *PLAAT4* mRNA levels in A549 cells transfected with *CSNK2B* versus control siRNAs for 72 h, followed by treatment with IRF1 agonists, all-trans retinoic acid (ATRA, 3 μM) or IFNγ (1000 U/ml) for 24 h. *P < 0.05, **P < 0.01 (*n* = 3, two-tailed Student's *t*-test). (**C**) Dose-dependent effects of IRF1 agonists on HAV/NLuc (18f/NLuc) in A549 cells. Drugs were added 4 h post-infection with HAV/NLuc at an m.o.i. of 1 and NLuc activity determined 72 h later. (**D**) Effects of depletion of *CSNK2B*, *IRF1* and *PLAAT4* on suppression of HAV/NLuc replication by IRF1 agonists, ATRA (1 μM) and IFNγ (1000 U/ml). LU, light units. *P < 0.05, **P < 0.01 versus control (*n* = 3, one-way ANOVA with Dunnett's multiple comparisons test). (**E**) Validation of the NLuc results by quantitation of HAV RNA levels using RT-qPCR. GEq, genome equivalents. *P < 0.05 versus control (*n* = 3, one-way ANOVA with Dunnett's multiple comparisons test). (**F**) Immunoblots of CSNK2B, IRF1, PLAAT4 and GAPDH as loading control in lysates of A549 cells transfected with indicated siRNAs. (**G**) Effects of depletion of either *CSNK2B* or *PLAAT4*, or both on suppression of HAV/NLuc replication by an IRF1 agonist, IFNγ (1000 U/ml). Knockdown efficiencies of each gene are shown on right. **P < 0.01, ***P < 0.0001 versus control (one-way ANOVA with Dunnett's multiple comparisons test).

The data presented above show CSNK2B positively regulates the basal activity of IRF1, thereby maintaining constitutive transcription of its target gene, *PLAAT4*, in both PH5CH8 cells and primary hepatocytes (Figures 1C and 4C). To further investigate its role in agonist-induced activation of IRF1, we examined the impact of *CSNK2B* depletion on *PLAAT4* expression following treatment with the IRF1 agonists, IFNγ and all-trans retinoic acid (ATRA), which induces IRF1 transcription in a STAT1-independent manner (21). Since ATRA failed to induce *PLAAT4* expression in PH5CH8 cells (Supplementary Figure S5A), we used A549 cells, in which there is dose-dependent ATRA induction of *PLAAT4* expression (Figure 5A; Supplementary Figure S5B). Consistent with a STAT1-independent activation of IRF1 transcription by ATRA, pre-treatment with pyridone 6 did not affect induction levels of both *IRF1* and *PLAAT4* (Supplementary Figure S5C). We confirmed that these agonist-induced responses were significantly attenuated upon *CSNK2B* depletion (Figure 5B). ATRA dose-dependently suppressed HAV replication by 50% at 1 μM or higher (Figure 5C), which correlated well with *PLAAT4* induction levels (Figure 5A). IFNγ was more efficient at inducing *PLAAT4* and impaired HAV replication at >300 U/ml (Figure 5C). The antiviral effects elicited by the IRF1 agonists were completely reversed or attenuated by silencing *CSNK2B or PLAAT4* (Figure 5D–F). We also examined anti-HAV effects of IFNγ in the context of depletion of either *CSNK2B* or *PLAAT4* versus simultaneous depletion of both genes (Figure 5G). While an additive increase in HAV replication was observed when both genes were simultaneously depleted, the increase caused by *CSNK2B* depletion was smaller in *PLAAT4*-depleted (1.99-fold) than control cells (8.18-fold). Together, these results attest to the functional importance of the CSNK2B-IRF1-PLAAT4 axis in the induced IRF1 response.

### Genome-wide landscape of IRF1-binding sites affected by CSNK2B

**Figure 6.**
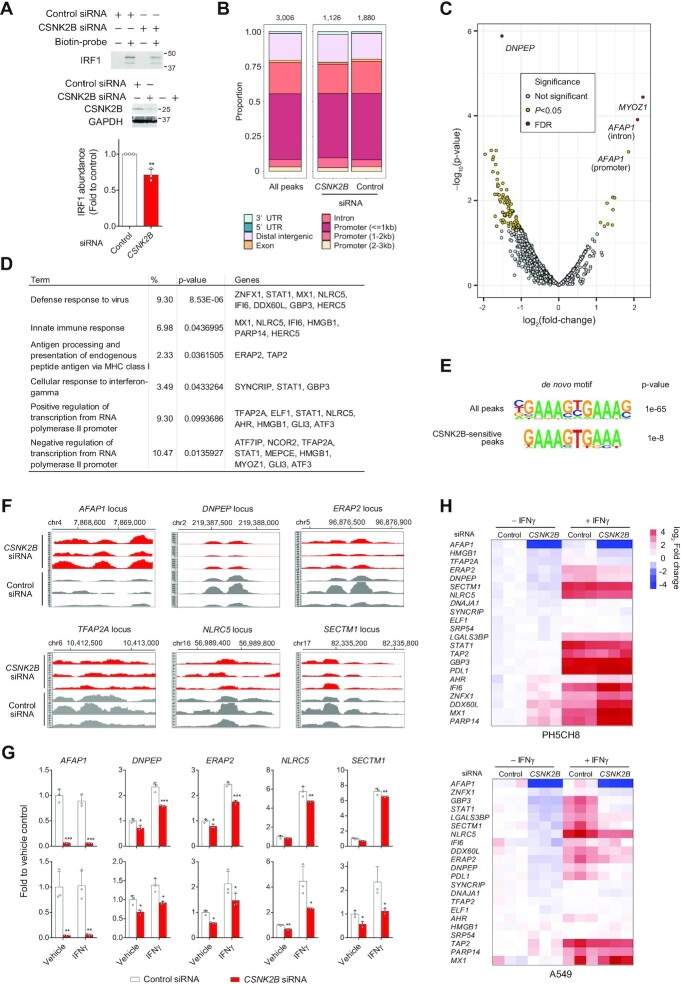
Genome-wide mapping of CSNK2B-regulated IRF1 binding sites by CUT&RUN. (**A**) In vitro pull-down experiments using biotinylated DNA probe containing IRF1 binding motifs derived from the *PLAAT4* promoter in lysates of *CSNK2B*-depleted PH5CH8 cells versus control (top). Means ± S.D. of relative values for abundance of DNA-bound IRF1 in *CSNK2B*-depleted cell lysates versus control (bottom). **P < 0.01 (*n* = 3, two-tailed Student's *t*-test). (**B**) Stacked bar plots showing peak annotation of IRF1-bound sites in PH5CH8 cells transfected with *CSNK2B* siRNA versus control. The number of peaks is shown on each of the stacked bar. (**C**) Volcano plots showing changes in relative abundance of the peaks identified in CUT&RUN between PH5CH8 cells transfected with *CSNK2B* versus control siRNAs. Each symbol represents the log_2_ fold-change in *CSNK2B* siRNA-transfected cells over control from three biological replicates. Combined *P*-value for the peak was determined using Sime's method. The *P*-values were adjusted using false discovery rate (FDR) correction. (**D**) Gene ontology analysis and functional annotation of genes differentially bound by IRF1 in *CSNK2B*-depleted cells. (**E**) Logo depiction of HOMER *de novo* motif analysis of all peaks (top) and peaks significantly decreased in *CSNK2B*-depleted cells (bottom). (**F**) Example genomic regions showing binding peaks of IRF1 in control versus *CSNK2B*-depleted PH5CH8 cells for all three biological replicates. (**G**) Bar graphs showing RT-qPCR measurements of transcripts from representative CSNK2B-responsive genes in PH5CH8 (upper panels) and A549 (lower panels) cell lines. Cells were stimulated with IFNγ (100 U/ml, + IFNγ) or vehicle control (− IFNγ) for 24 h. *P < 0.05, **P < 0.01, ***P < 0.0001 (*n* = 3, two-way ANOVA with Sidak's multiple comparisons test). (**H**) Heatmaps showing the relative abundance of CSNK2B-regulated gene transcripts linked to antiviral defense and innate immune responses as determined by RT-qPCR, with clustering according to CSNK2B regulation of basal expression.

While our results show CSNK2B enhances *PLAAT4* transcription, it remains uncertain how it does so. To examine whether CSNK2B modulates the binding affinity of IRF1 to the *PLAAT4* promoter element, we conducted *in vitro* pull-down experiments using a biotinylated DNA probe containing the IRF1 binding motif derived from the *PLAAT4* promoter and compared the IRF1 abundance co-precipitated with the DNA probe in *CSNK2B*-depleted PH5CH8 cell lysates versus control. Whereas CSNK2B by itself did not bind a DNA probe containing the IRF1 binding motifs, IRF1 binding was reduced in *CSNK2B*-depleted lysates (Figure 6A). These data suggest CSNK2B binding to IRF1 enhances its affinity for the *PLAAT4* promoter. Thus, we hypothesized that CSNK2B might similarly modulate the affinity of IRF1 binding to other IRF1-response elements, thereby enhancing transcription of many IRF1 target genes. To gain a comprehensive view of CSNK2B-dependent IRF1 binding affinity across the genome, we applied CUT&RUN technology, a recently developed method to profile protein-bound chromatin in which antibody-guided cleavage by protein A/G-fused micrococcal nuclease releases protein-DNA complexes that are subsequently subjected to high-throughput DNA sequencing (22). CUT&RUN experiments were carried out using an IRF1 antibody and PH5CH8 cells transfected with either *CSNK2B*-specific or control non-targeting siRNAs. Analysis of genome-wide IRF1 occupancy patterns revealed a high enrichment of promoter-proximal regions of IRF1-regulated genes (Figure 6B; Supplementary Figure S6A), with greater quantities of annotated peaks isolated from cells transfected with control siRNA (626.67 ± 177.99 S.E.M.) relative to *CSNK2B*-depleted cells (375.33 ± 69.67 S.E.M.). Despite large variations across the biological replicates analyzed by CUT&RUN, differential binding analysis showed numerous sites where IRF1 binding affinity is significantly changed, with the majority weakened in the absence of CSNK2B (Figure 6C; Supplementary Table S2). Differentially bound regions found in the experiments contain loci of genes classified in diverse functional categories, including antiviral responses, innate immune responses, and transcriptional control (Figure 6D). *De novo* motif analysis of IRF1-bound DNA sequences using HOMER showed an enrichment in IRF1 regulatory motifs. While the motifs identified in both *CSNK2B*-depleted and control cells showed a typical IRF1 consensus (GAAA)NN(GAAA) sequence, those found in the peaks significantly reduced by *CSNK2B* depletion were highly constrained to ‘(GAAA)GT(GAAA)’ (Figure 6E). To assess whether CSNK2B selectively modulates IRF1 binding to different promoter sequences, we used NLuc reporter constructs containing 4 tandem repeats of the ‘GAAA’ segment with ‘GT’, ‘GC’ or ‘GA’ linker dinucleotides (Supplementary Figure S6B). While the constructs with ‘GT’ and ‘GC’ possessed strong promoter activity, that with ‘GA’ was 20-fold less active, showing its weaker affinity to endogenous IRF1 (Supplementary Figure S6B). However, each of these reporters was repressed by about 50% upon CSNK2B depletion (Supplementary Figure S6C), indicating that the selectivity of the CSNK2B-IRF1 complex is not determined solely by the linker sequences.

To determine how altered IRF1-binding affects the basal and agonist-induced transcription in *CSNK2B*-depleted cells, we conducted quantitative RT-qPCR analysis of genes with promoter occupancy regulated in a highly significant manner (*AFAP1* and *DNPEP*) and those involved in antiviral responses (*DDX60L*, *ELF1*, *ERAP2*, *HMGB1*, *LGALS3BP*, *MX1*, *STAT1* and *ZNFX1*), proviral factors (*AHR*, *DNAJA1*, *SRP54* and *TFAP2*), and immune responses (*DNPEP*, *PARP14*, *SECTM1*, *SYNCRIP* and *TAP2*) in unstimulated and IFNγ-treated PH5CH8 cells. With few exceptions, most genes were suppressed basally and less well induced by IFNγ when depleted of *CSNK2B* (Figure 6F, G, H), mirroring the effect on *PLAAT4* (Figure 5B). The impact of CSNK2B in enhancing IRF1-bound genes was even more pronounced in A549 cells (Figure 6G, H). However, none of the genes active in suppressing the replication of Flaviviridae (*APOL1* and *PSMB9*) were altered (Supplementary Table S2) (3). Consistent with this, *CSNK2B* depletion did not enhance replication of hepatitis C virus, even when replication levels were markedly boosted by the pan-JAK inhibitor (Supplementary Figure S7). These results suggest CSNK2B affects numerous IRF1 effector genes representing diverse functional categories, but that it regulates only a subset of IRF1-regulated antiviral genes due to selectivity of the IRF1-CSNK2B complex.

### Loss of CSNK2B leads to abnormal accumulation of IRF1 at the *AFAP1* loci and impairs its function

**Figure 7.**
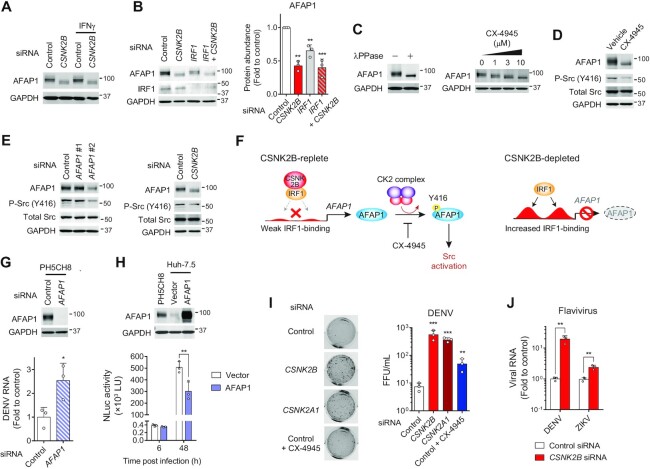
CSNK2B regulates transcription and phosphorylation of AFAP1 that lowers permissiveness to flavivirus replication. (**A**) Immunoblots of AFAP1 and GAPDH as loading control in lysates of PH5CH8 cells transfected with indicated siRNAs, with and without IFNγ stimulation. (**B**) Immunoblots of AFAP1, IRF1 and GAPDH in lysates of PH5CH8 cells depleted of either (or both) CSNK2B and IRF1. Quantitation of AFAP1 protein abundance is shown on the right. **P < 0.01, ***P < 0.0001 (*n* = 3, one-way ANOVA with Dunnett's multiple comparisons test). (**C**) Immunoblots of AFAP1 and GAPDH in lysates of PH5CH8 cells treated with either lambda protein phosphatase (λ PPase, left panels) or indicated concentrations of CX-4945 (right panels). (**D**) Immunoblots of AFAP1 and Src in lysates of PH5CH8 cells treated with 10 μM CX-4945. (**E**) Immunoblots of PH5CH8 cell lysates transfected with indicated siRNAs targeting *AFAP1* (left panels) or *CSNK2B* (right panels). (**F**) Scheme of CSNK2B-regulated AFAP1 signaling cascades that lead to Src activation. (**G**) Immunoblots of PH5CH8 cell lysates transfected with indicated siRNAs (top). DENV RNA levels were determined at 48 h p.i. (bottom). *P < 0.05 (*n* = 3, two-tailed Student's *t*-test). (**H**) Immunoblots of AFAP1 in lysates of PH5CH8 versus Huh-7.5 cells stably transduced with AFAP1 (top). Huh-7.5 cells stably expressing AFAP1 or vector control were challenged with DENV/NLuc (bottom). NLuc activities at the indicated time points post-infection are shown. **P < 0.01 (*n* = 3, two-way ANOVA with Sidak's multiple comparisons test). (**I**) PH5CH8 cells were transfected with indicated siRNAs and infected 48 h later with dengue virus (DENV) at an m.o.i. of 0.1. CX-4945 (5 μM) was added 2 h post-infection. Infectious titers were determined 48 h p.i. by focus formation assays. FFU, focus forming units. **P < 0.01, ***P < 0.0001 versus control (*n* = 3, one-way ANOVA with Dunnett's multiple comparisons test). (**J**) PH5CH8 cells were infected with DENV or Zika virus (ZIKV) as in (I) and viral RNA levels determined by RT-qPCR. **P < 0.01 (*n* = 3, two-tailed Student's *t*-test).

Unexpectedly, we observed that *CSNK2B* depletion resulted in impaired expression of *AFAP1* (actin filament-associated protein 1) (Figure 6G, H), despite increased IRF1 binding to both promoter and intron sites within the gene (Figure 6C, F). Thus, CSNK2B can modulate gene expression as a consequence of altered IRF1-binding to the DNA (Figure 6G, H). Immunoblot analysis confirmed the low-abundance and faster migrating band of AFAP1 at the protein level in *CSNK2B*-depleted cells (Figure 7A). Whereas abnormal accumulation of IRF1 at the *AFAP1* loci impairs *AFAP1* transcription (Figure 6F, G), absence of IRF1 lead to reduced AFAP1 expression, indicating that the weak binding of IRF1 is still required for optimal transcription of *AFAP1* (Figure 7B). We hypothesized that the faster migrating band detected in *CSNK2B*-depleted cells might be caused by an alternative transcription start site resulting from massive IRF1 binding at the promoter and intron sites, which could possibly generate an alternative transcript encoding a shorter open reading frame. However, the sequences of *AFAP1* transcripts from *CSNK2B*-depleted and control cells were both identical to transcript variant 3 (GenBank ID: NM_001371090). Thus, we explored an alternative hypothesis, namely that the more rapidly migrating bands represent decreased CK2-catalyzed phosphorylation of AFAP1. In support of this, λ phosphatase treatment, as well as pharmacologic inhibition of CK2, converted the immunoreactive bands into a single faster migrating band (Figure 7C). These results show that CK2 controls AFAP1 at both transcription and post-translational levels.

AFAP1 is an adaptor molecule that modulates actin filament integrity and activates Src signaling in response to cellular signals (23,24). Increased *AFAP1* abundance is an unfavorable prognostic marker in lung and renal cancers (25) and promotes tumorigenesis of prostate cancer (26). In contrast to prior studies in which AFAP1 was activated by PKC-regulated phosphorylation (26,27), we found that pharmacological inhibition of CK2 is sufficient to abrogate AFAP1 phosphorylation and prevent expression of the activated form of Src (phosphorylation at Y416) (Figure 7D). siRNA depletion of CSNK2B phenocopied this reduction in Src activation (Figure 7E). Thus, CSNK2B-initiated signals to activate transcription and phosphorylation of AFAP1 extends to downstream Src activation (Figure 7F).

Lastly, we assessed how AFAP1 crosslinked to the actin cytoskeleton may affect viral replication downstream of IRF1 and CSNK2B. While some classes of viruses do not require the host cell cytoskeleton for replication (28), the replication of flaviviruses relies on remodeling of the actin cytoskeleton (29) and is potently restricted by IRF1 (1,3). We found that siRNA-mediated depletion of *AFAP1* increased permissiveness to dengue virus (DENV) replication in PH5CH8 cells (Figure 7G), suggesting an antiviral effector function. Moreover, only low expression of AFAP1 protein was found in highly permissive Huh-7.5 cells. Ectopic expression of AFAP1 in Huh-7.5 cells reduced DENV replication overall, although it was without effect at an early time point postinfection (∼6 h) (Figure 7H). Importantly, depletion of *CSNK2B* as well as a catalytic subunit (*CSNK2A1*) of CK2 resulted in a 50-fold increase in production of infectious DENV particles in PH5CH8 cells (Figure 7I). Pharmacologic inhibition of CK2 had relatively small effects in enhancing DENV production, likely due to the cytotoxicity of the compound (Figure 7I). *CSNK2B* depletion also enhanced viral RNA levels in both DENV and Zika virus-infected cells (Figure 7J). Thus, AFAP1 mediates at least part of the potent anti-flavivirus activities of the CSNK2B-IRF1 complex and CK2.

## DISCUSSION

We show here that CSNK2B is an important regulator of both ‘basal’ and ‘agonist-induced’ activity of IRF1. In contrast to a prior *in vitro* biochemical study suggesting that CSNK2A1 might phosphorylate and activate IRF1 (5), our results based on Phos-tag gel analysis (Figure 2D, E) and site-directed mutagenesis of candidate phosphorylation residues in IRF1 (Supplementary Figure S3C, D) showed that CSNK2B modulates IRF1 activity independently of CK2-mediated phosphorylation of IRF1 protein. While pharmacological inhibition of CK2 caused opposing effects on IRF1 function in Huh-7.5 cells versus PH5CH8 cells, our data suggest differential regulation of CSNK2B abundance in response to the drug underlies the functional differences between these cell lines (Figure 2G, H, I, J; Supplementary Figure S2). The mechanism of CSNK2B action remains incompletely explained, but we show CSNK2B generally enhances IRF1 binding at specific promoter sites (Figure 6C, F). We hypothesize that this is due to a CSNK2B-induced change in IRF1 conformation. This model is supported by CUT&RUN experiments that show depleting *CSNK2B* results in reduced binding of IRF1 at numerous sites in the genome, while at a few sites the absence of CSNK2B paradoxically enhances DNA binding, as exemplified by increased accumulation of IRF1 at *AFAP1* loci in *CSNK2B*-depleted cells (Figure 6C, F). Overall, these results suggest that loss of CSNK2B leads generally to reduced transcription of IRF1-regulated genes. However, not all IRF1 targets were found to be CSNK2B-dependent, implying a complexity of IRF1 interactions with genomic DNA. The molecular and structural basis underlying the selectivity of the CSNK2B-IRF1 complex needs to be clarified in future studies. IRF1 typically regulates gene transcription by binding to promoter sites, but our CUT&RUN experiments also revealed numerous examples of IRF1 binding at non-promoter sites (e.g. introns and untranslated regions) within the locus of genes that are not expressed, such as *MYOZ1* (Figure 6C; Supplementary Table S2). This suggests IRF1 might have additional functions in modulating chromatin structure beyond conventional transcriptional activity.

Consistent with the pan-viral activity of IRF1 that restricts a broad range of viruses (1), depletion of CSNK2B promoted replication of multiple viruses, including the picornavirus HAV and flaviviruses, DENV, and ZIKV (Figures 1D, E, F, 4D, 7I, J). While the inhibitory effect of CSNK2B against HAV is likely due to its ability to promote *PLAAT4* transcription (Figures 3, 4C), the primary IRF1-regulated anti-*Flaviviridae* effectors, *APOL1* and *PSMB9* (1,3), were not affected by silencing *CSNK2B*. By contrast, our CUT&RUN analysis newly identified AFAP1 as an anti-flavivirus effector transcriptionally regulated by the CSNK2B-IRF1 complex (Figure 6C, F, G, H). It is noteworthy that CSNK2B also mediates phosphorylation-dependent activation of AFAP1 (Figure 7D, E), which contributes to the potent antiviral effects elicited by CK2 (Figure 7I, J).

Despite the potent antiviral activities of CSNK2B revealed in this study, the antiviral nature of the protein has only been reported previously for influenza A virus entry (30). In stark contrast to its role as an antiviral factor, CSNK2B as a component of CK2, has been suggested to be a pro-viral cofactor that phosphorylates and activates viral proteins of several important pathogens, such as HCV, human immunodeficiency virus, respiratory syncytial virus, and herpesviruses (31). Thus, independently of the role of CSNK2B as an IRF1 cofactor, disparate effects of the CK2 complex on different viruses likely determine viral phenotype resulting from *CSNK2B* depletion. Of note, the nucleocapsid (N) protein of SARS-CoV-2 directly interacts with CK2 components (CSNK2A2 and CSNK2B) and activates its catalytic activity for productive infection (32), and pharmacological intervention of these key processes with CIGB-325, an anti-CK2 peptide, has been shown to attenuate pulmonary lesions in COVID-19 patients in a randomized clinical trial (33). While promising, the contrasting effects of CK2 on different viruses highlight the need for careful monitoring of coinfections with CK2-sensitive pathogens (e.g. flaviviruses) when considering the use of CK2 inhibitors as anti-pathogen therapy.

In addition to host anti-pathogen defense, IRF1 has been recognized to play important but opposing roles in the regulation of tumor progression. On one hand, IRF1 drives escape from anti-tumor immune surveillance by binding directly to the promoter of *PD-L1*, a ligand of the programmed death-1 (PD-1) immune checkpoint, thereby promoting its expression [114]. On the other hand, genetic loss of *IRF1* is frequently found in various human cancers, including leukemia and gastric cancers (34,35). The tumor suppressor nature of IRF1 has been linked to its pro-apoptotic property, and it acts as an effector of IFNs that suppress proliferation in many cancers (36). Yet, cellular signaling pathways that mediate pro- and anti-tumor effects of IRF1 are not fully elucidated. The data presented here show AFAP1, an actin filament-associated protein implicated in promotion of tumorigenic growth (26,37), to be an important effector regulated by IRF1, accumulation of which at the *AFAP1* loci is fine-tuned by CSNK2B (Figure 6F). In addition to its function in activating *AFAP1* transcription, CSNK2B acts as a CK2 complex to promote AFAP1 phosphorylation that subsequently activates Src signaling (Figure 7D, E). Importantly, elevated expression of CK2 is frequently found in cancers, suggesting that the oncogenic nature of this enzyme may be a feasible molecular target for treatment of chemoresistant tumors (38,39). Several cellular processes have been proposed for oncogenic actions driven by CK2 (40), such as Wnt/β-catenin, PTEN/Akt/mTORC1 and HIF-1 signaling pathways. It will be important to determine how the CK2-AFAP1-Src axis interacts with these pathways.

Our results show a new role for AFAP1 as a downstream antiviral effector of IRF1 and CSNK2B/CK2 signaling that down-regulates a late step in the replication of DENV. Whereas AFAP1 is tightly crosslinked to actin filament and controls its integrity, loss- and gain-of-function experiments confirmed that expression of AFAP1 is associated with reduced permissiveness to DENV replication (Figure 7G, H). It seems likely that actin-bound AFAP1 may prevent the virus-induced cytoskeletal rearrangements observed in DENV infection (41,42), thereby down-regulating the formation of virus replication factories (43).

In summary, our results reveal that the DNA-binding activity of IRF1 is regulated post-translationally by CSNK2B, and suggest that the transcriptional activity of IRF1 in different tissues may be influenced by the expression status of CSNK2B. While the target selectivity of IRF1-CSNK2B complex remains to be elucidated, further clarifying the mechanisms by which CSNK2B modulates IRF1 binding to its target DNA elements likely provide the basis for developing antiviral and anticancer therapies.

## DATA AVAILABILITY

ChIP-Seq data generated in this study are available in the SRA database under the accession code PRJNA885123.

## Supplementary Material

gkad298_Supplemental_Files
