# Supplementary material for: CSNK2B modulates IRF1 binding to functional DNA elements and promotes basal and agonist-induced antiviral signaling
Source: Nucleic Acids Res. 2023 Apr 24;51(9):4451–66. doi: 10.1093/nar/gkad298 (PMC10201418; doi:10.1093/nar/gkad298)
Supplement: gkad298_Supplemental_Files [file gkad298_supplemental_files.zip › Supplementary Data_Rev2.pdf]

A

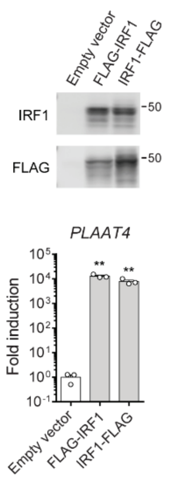

B

Classification summary	Gene
Response to DNA damage stimulus	BCLAF1, PARP1, PRKDC, XRCC5, XRCC6
Spliceosome	SRRM2, SF3B5, RBM17, SF3B2, DDX5, HNRNPA3, HNRNPU, SYNCRIP, HNRNPM, HNRNPK, SNRPD2, SNRPD1, HNRNPH1, HNRNPA2B1, PABPC1, SNRPD3, HNRNPC, HNRNPA1, SF3B1, NCL
Ribonucleoprotein	RPL4, RPL5, RPL30, RPL3, RPL32, RPL31, RPL34, RPLP1, HNRNPU, RPL10A, RPL8, RPL9, RPL7, RPL39P5, RPS4X, RPS15, RPS14, RPL7A, SYNCRIP, RPS17, RPS16, RPS19, SNRPD2, RPL18A, RPS18, SNRPD1, RPL36, RPLP2, RPL35, RPL38, SNRPD3, RPS11, RPS13, RPS12, RPS9, SNRPN, RPL21, RPS7, RPS8, RPL23, RPS6, SRP9, DKC1, HNRNPH1, RPL24, RPL27, RPL26, RPL29, RPL28, RPL10, RPL12, RPS15A, RPS3, RPL14, RPL13, RPS2, RPL15, RPS27A, HNRNPA1, RPL18, RPL17, HNRNPA0, HNRNPA3, HNRNPL, HNRNPM, RPS25, RPS27, HNRNPK, RPS29, RPL27A, HNRNPA2B1, RPS26P11, HNRNPD, RPS20, FAU, HNRNPC, RPS24, RPS23
Chromatin	XRCC6, HIST1H4A, XRCC5, HIST1H1E, HIST3H2BB, H1FX, BANF1, HIST1H2AA, NCL
Proteasome	PSMA7, PSMA5, PSMA6, PSMA3, PSMD7, PSMB5, PSMD4, PSMA1, PSMB2, PSMC4, PSMA2, PSMD2, PSMB1
Chaperone	HSPA9, DNAJA1, HSP90AA1, SET, HSP90AB1, CCT5
Stress response	HSP90AA1, HSPH1, HSP90AB1, HSPA4
Nucleus	RPL5, RPL3, HNRNPU, SYNCRIP, RPS19, SNRPD2, SNRPD1, C1QBP, CFL1, HIST1H1E, HIST3H2BB, BANF1, SNRPD3, HIST1H2AA, EIF5AL1, DDX17, SRRM2, RBM17, SNRPN, PARP1, NME2, ILF2, KCTD5, HCFC1, PSMA5, PSMA6, ILF3, PSMA3, PSMA1, PSMA2, DKC1, HNRNPH1, CLNS1A, IRF1, NCL, MATR3, PABPC1, SF3B5, SF3B2, DDX5, SET, DDX21, PSMA7, BCLAF1, PSMB5, PSMB2, PSMB1, IGF2BP1, RPS3, H1FX, RPS27A, HNRNPA1, SF3B1, HDX, E2F7, HNRNPA0, HSPA9, HNRNPA3, XRCC6, XRCC5, HNRNPAB, DNAJA1, HNRNPL, RSL1D1, HNRNPM, HIST1H4A, HNRNPK, PSMC4, HNRNPA2B1, HNRNPD, HNRNPC, COPS8, OGT
Regulation of NF-kappaB activity	TAB1, TAB3, MAP3K7, PRDX1
Regulation of transcription	BCLAF1, HCFC1, NCL, NME2

Supplementary Figure S1. Validation of functional expression of FLAG-tagged IRF1 and gene ontology classification of cellular proteins co-precipitated with affinity-purified IRF1 proteins. **(A)** Immunoblots showing expression of FLAG-tagged IRF1 in transfected 293FT cells. Induction of a IRF1-regulated gene, *PLAAT4*, by the ectopically expressed IRF1 was validated by RT-qPCR analysis. $^{**}P < 0.01$ versus empty vector control (n = 3, one-way ANOVA with Dunnett's multiple comparisons test). **(B)** Functional annotation of IRF1-associated proteins. Gene ontology enrichment analysis was performed using DAVID 6.8.

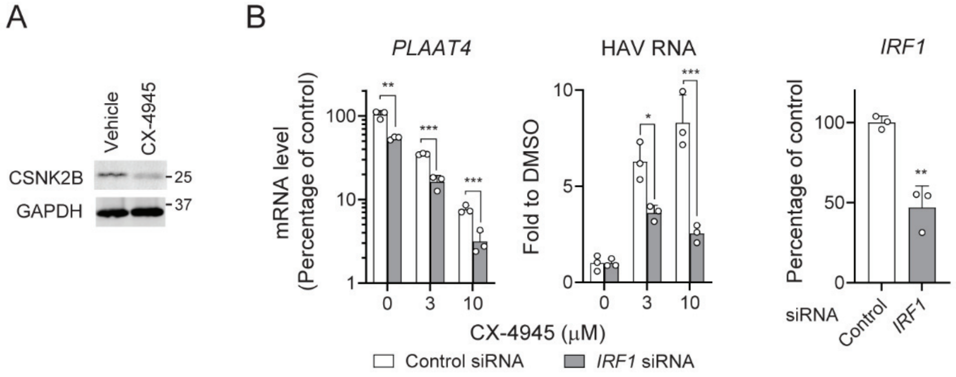

Supplementary Figure S2. Effects of CX-4945 treatment on CSNK2B abundance and IRF1-regulated restriction of HAV replication in PH5CH8 cells. (A) Immunoblots showing CSNK2B protein abundance in PH5CH8 cells treated with 10 μM CX-4945 for 24 h. **(B)** Effects of IRF1 depletion on CX-4945-induced regulation of *PLAAT4* and HAV replication in PH5CH8 cells. $^{*}P < 0.05$, $^{**}P < 0.01$, $^{***}P < 0.0001$ versus control (n = 3, two-way ANOVA with Sidak's multiple comparisons test).

A

Phospho site	Peptide detected
S87	CAMNS[Pho]LPDIEEVKDQSR
T266	GYLLNEPGVQPT[Pho]SVYGDFSCK
S273	GYLLNEPGVQPTSVYGDFS[Pho]CKEEPEIDSPGGDIGLSLQR
S282	EEPEIDS[Pho]PGGDIGLSLQR
T311	NMDATWLDSLLT[Pho]PVR

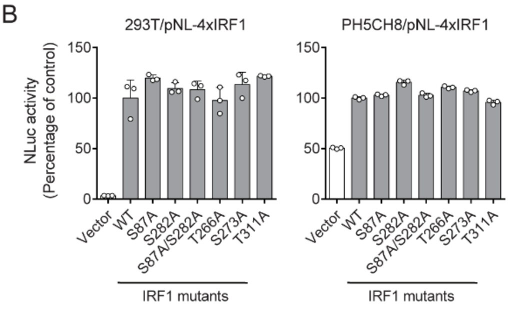

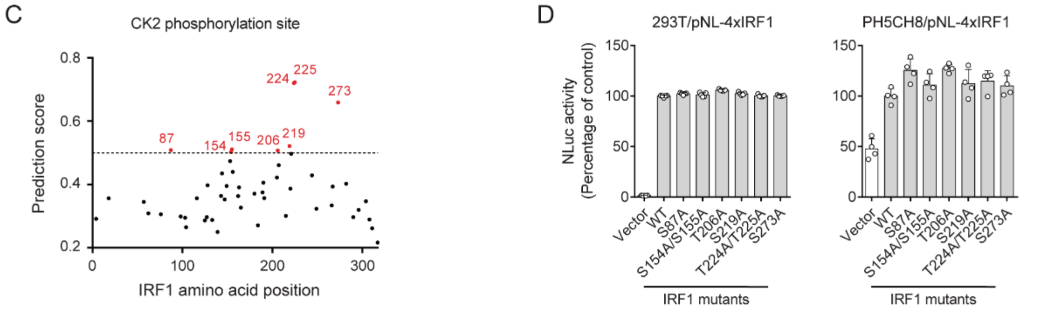

Supplementary Figure S3. Alanine scanning mutagenesis of candidate phosphorylation sites within IRF1 and characterization of the mutants to activate transcription. (A) Identification of phosphorylated peptides derived from IRF1 by mass spectrometric analysis of affinity-purified IRF1 expressed in 293FT cells. **(B)** NLuc reporter analysis in two different cell lines (293T and PH5CH8) stably transfected with pNL-4×IRF1-NLuc. Cells were transfected with indicated IRF1 mutants and relative NLuc activity (mean ± S.D.) at 24 h post-transfection is shown. **(C)** NetPhos 3.1 prediction of CK2 phosphorylation sites in IRF1. Amino acid residues with the score of 0.5 or higher are shown in red. **(D)** NLuc reporter assay was carried out using indicated IRF1 mutants as in **(B)**. Values are shown as mean ± S.D.

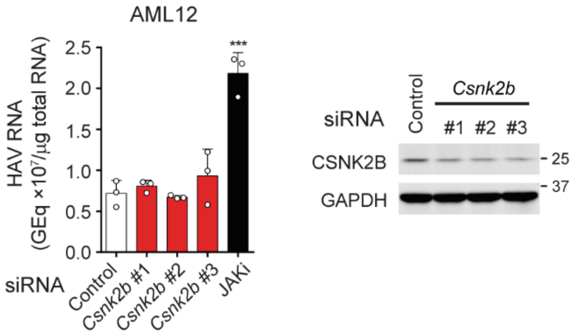

Supplementary Figure S4. Absence of CSNK2B effects in murine hepatocytes. AML12 cells were transfected with indicated siRNAs and infected 48 h later with hepatitis A virus (HAV) at an m.o.i. of 10. Percentage of HAV RNA levels was determined 4 d p.i. by RT-qPCR (left panel). Knockdown efficiency was determined 2 d post-siRNA transfection by Western blotting analysis (right panel). JAKi, 300 nM pyridone 6. $^{***}P < 0.0001$ ($n = 3$, one-way ANOVA with Dunnett's multiple comparisons test).

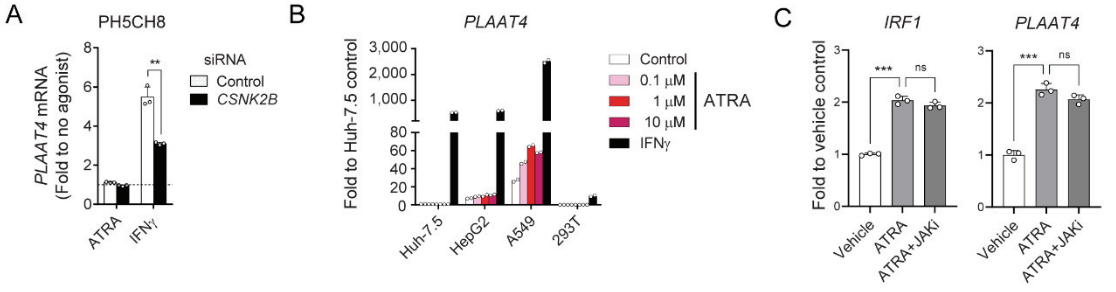

Supplementary Figure S5. Agonist-induced expression of *PLAAT4* in different cell lines. (A) RT-qPCR determination of *PLAAT4* mRNA levels in PH5CH8 cells transfected with *CSNK2B* versus control siRNAs for 72 h, followed by treatment with IRF1 agonists, all-trans retinoic acid (ATRA, 10 μM) or IFNγ (1,000 U/mL) for 24 h. $^{**}P < 0.01$ (n = 3, two-way ANOVA with Sidak's multiple comparisons test). **(B)** *PLAAT4* mRNA levels in different cell lines treated with indicated doses of ATRA or IFNγ (1,000 U/mL) for 24 h (n = 2). **(C)** Effects of a pan-JAK inhibitor (JAKi, pyridone-6) on ATRA-induced transcription of *IRF1* and *PLAAT4*. PH5CH8 cells were pretreated with 300 nM pyridone 6 for 30 min and then incubated with 1 μM ATRA in the continued presence of pyridone 6 for 24 h. *IRF1* and *PLAAT4* transcript levels were determined by RT-qPCR analysis. $^{***}P < 0.0001$ (n = 3, one-way ANOVA with Dunnett's multiple comparisons test).

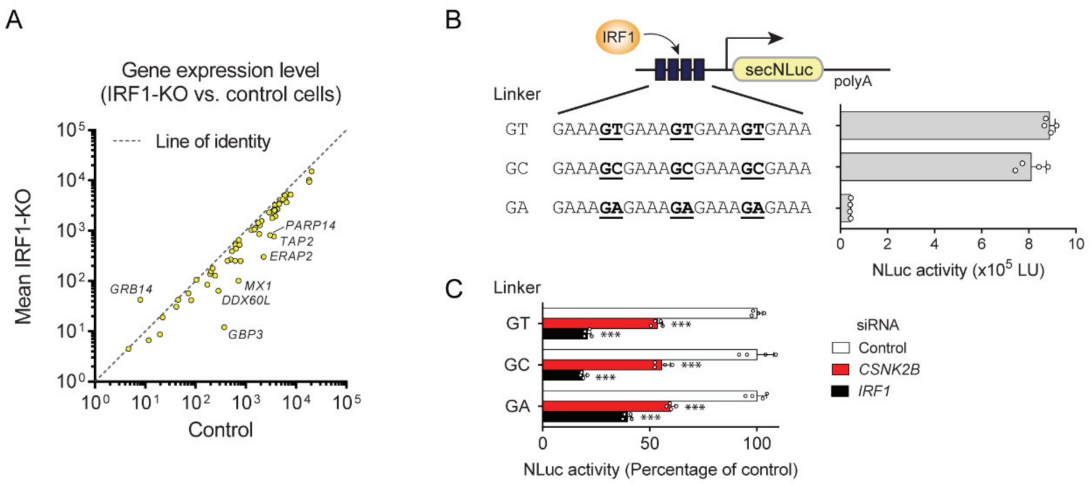

Supplementary Figure S6. Characterization of IRF1-bound genes identified in CUT&RUN and the DNA sequence that binds with high affinity to endogenous IRF1. (A) Expression profiles of genes regulated by CSNK2B-IRF1 complex in IRF1-KO cells versus control as determined by a re-analysis of previously published data (GEO: GSE114916). Note that all genes, except for *GRB14*, were down-regulated in IRF1-KO cells. **(B)** DNA composition-dependent activity of IRF1. PH5CH8 cells were transiently transfected with modified NLuc reporter constructs as indicated and NLuc activity at 24 h post-transfection is shown. LU, light units. **(C)** PH5CH8 cells were transfected with siRNAs targeting *CSNK2B*, *IRF1* or control, and re-transfected with indicated NLuc reporter DNAs 48 h later. Relative NLuc activity at 24 h post DNA transfection is shown. $^{***}P < 0.0001$ versus control (n = 3, two-way ANOVA with Dunnett's multiple comparisons test).

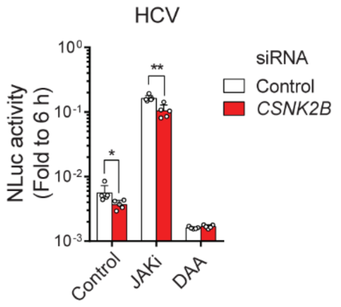

Supplementary Figure S7. Effects of *CSNK2B* depletion on replication of HCV. (A) PH5CH8 cells were co-electroporated with HCV RNA (JFH1-QL/NLuc, 5 µg), 250 nmol miR-122 duplex mimics, and 250 nmol siRNA. NLuc produced from PH5CH8 cells transfected with control versus *CSNK2B* siRNAs at 72 h post RNA electroporation is shown. JAKi, 300 nM pyridone 6; DAA, 30 µM Sofosbuvir. $^{*}P < 0.05$, $^{**}P < 0.01$ (n = 5, two-tailed Student's t-test).

Supplementary Table S1. List of proteins detected by MS

(A) Proteins found in empty vector control

#	Uniprot ID#	Name	Gene name	Peptides (95%)	% Cov (95)
1	P23588	Eukaryotic translation initiation factor 4B	EIF4B	12	18
2	P52732	Kinesin-like protein KIF11	KIF11	9	7.8
3	Q01804	OTU domain-containing protein 4	OTUD4	6	5
4	O75688	Protein phosphatase 1B	PPM1B	6	12.9
5	P08107	Heat shock 70 kDa protein 1A/1B	HSPA1A	6	9.8
6	P63261	Actin, cytoplasmic 2	ACTG1	8	13.9
7	O14744	Protein arginine N-methyltransferase 5	PRMT5	6	5.5
8	Q9Y657	Spindlin-1	SPIN1	4	15.3
9	P98175	RNA-binding protein 10	RBM10	2	2.3
10	P61247	40S ribosomal protein S3a	RPS3A	3	8.7
11	Q02878	60S ribosomal protein L6	RPL6	2	8.3
12	Q15208	Serine/threonine-protein kinase 38	STK38	1	1.7
13	Q9Y2W1	Thyroid hormone receptor-associated protein 3	THRAP3	1	0.7
14	Q99417	C-Myc-binding protein	MYCBP	1	9.7
15	P11142	Heat shock cognate 71 kDa protein	HSPA8	2	2.9
16	Q9ULV4	Coronin-1C	CORO1C	1	2.3
17	Q9BRS2	Serine/threonine-protein kinase RIO1	RIOK1	1	2.1
18	Q9BQA1	Methylosome protein 50	WDR77	1	3.8
19	Q969Q0	60S ribosomal protein L36a-like	RPL36AL	1	7.5
20	P84090	Enhancer of rudimentary homolog	ERH	2	6.7
21	P62750	60S ribosomal protein L23a	RPL23A	1	7.1
22	Q9BUA3	Uncharacterized protein C11orf84	C11orf84	1	2.9
23	Q8WWY3	U4/U6 small nuclear ribonucleoprotein Prp31	PRPF31	1	2.2
24	Q8NHW5	60S acidic ribosomal protein	RPLP0P6	1	3.5
25	P60174	Triosephosphate isomerase	TPI1	1	5.2
26	P06748	Nucleophosmin	NPM1	1	4.4
27	P61604	10 kDa heat shock protein, mitochondrial	HSPE1	1	11.8
28	P67809	Nuclease-sensitive element-binding protein 1	YBX1	1	4.9

(B) Proteins associated with FLAG-IRF1

#	Uniprot ID#	Name	Gene name	Peptides (95%)	% Cov (95)
1	P10914	Interferon regulatory factor 1	IRF1	70	54.8
2	Q13813	Spectrin alpha chain, brain	SPTAN1	13	7.6
3	P19338	Nucleolin	NCL	10	15.4
4	P09874	Poly [ADP-ribose] polymerase 1	PARP1	9	11.1
5	P31943	Heterogeneous nuclear ribonucleoprotein H	HNRNPH1	9	23.2
6	Q01082	Spectrin beta chain, brain 1	SPTBN1	7	3.2
7	Q9Y2H1	Serine/threonine-protein kinase 38-like	STK38L	11	20
8	P09651	Heterogeneous nuclear ribonucleoprotein A1	HNRNPA1	6	24.7
9	P16989	DNA-binding protein A	CSDA	6	17.7
10	P51610	Host cell factor 1	HCFC1	5	3.3
11	P12956	X-ray repair cross-complementing protein 6	XRCC6	6	13
12	Q7Z353	Highly divergent homeobox	HDX	5	8.6
13	P10412	Histone H1.4	HIST1H1E	5	16.9
14	P13010	X-ray repair cross-complementing protein 5	XRCC5	5	7
15	Q9NYF8	Bcl-2-associated transcription factor 1	BCLAF1	4	6.5
16	Q9NXV2	BTB/POZ domain-containing protein KCTD5	KCTD5	4	25.2
17	Q9NYL9	Tropomodulin-3	TMOD3	4	16.2
18	Q06830	Peroxiredoxin-1	PRDX1	4	29.7
19	Q8WWY3	U4/U6 small nuclear ribonucleoprotein Prp31	PRPF31	5	9
20	P11940	Polyadenylate-binding protein 1	PABPC1	4	8.8
21	O60506	Heterogeneous nuclear ribonucleoprotein Q	SYNCRIP	3	7.4
22	Q86V81	THO complex subunit 4	THOC4	4	23.7
23	O15294	UDP-N-acetylglucosamine--peptide N-acetylglucosaminyltransferase 110 kDa subunit	OGT	3	4.3
24	P62314	Small nuclear ribonucleoprotein Sm D1	SNRPD1	4	37.8
25	P14866	Heterogeneous nuclear ribonucleoprotein L	HNRNPL	3	7
26	Q9NR30	Nucleolar RNA helicase 2	DDX21	3	5.1
27	P62316	Small nuclear ribonucleoprotein Sm D2	SNRPD2	4	32.2
28	Q9BQE3	Tubulin alpha-1C chain	TUBA1C	3	9.4
29	Q9NZI8	Insulin-like growth factor 2 mRNA-binding protein 1	IGF2BP1	3	6.6
30	P05141	ADP/ATP translocase 2	SLC25A5	3	10.1
31	Q99729	Heterogeneous nuclear ribonucleoprotein A/B	HNRNPAB	3	8.1
32	O75533	Splicing factor 3B subunit 1	SF3B1	2	2.2
33	Q9UQ35	Serine/arginine repetitive matrix protein 2	SRRM2	2	1.3
34	P52272	Heterogeneous nuclear ribonucleoprotein M	HNRNPM	2	5.2
35	P22626	Heterogeneous nuclear ribonucleoproteins A2/B1	HNRNPA2B1	2	7.6
36	Q5VTE0	Putative elongation factor 1-alpha-like 3	EEF1AL3	4	7.6
37	P61978	Heterogeneous nuclear ribonucleoprotein K	HNRNPK	2	6
38	Q96QV6	Histone H2A type 1-A	HIST1H2AA	2	12.2

39	Q5W0B1	RING finger protein 219	RNF219	2	4
40	Q15750	TGF-beta-activated kinase 1 and MAP3K7-binding protein 1	TAB1	2	4.2
41	O15355	Protein phosphatase 1G	PPM1G	2	6.2
42	P28074	Proteasome subunit beta type-5	PSMB5	2	9.9
43	Q8N5C8	TGF-beta-activated kinase 1 and MAP3K7-binding protein 3	TAB3	2	5.1
44	P07910	Heterogeneous nuclear ribonucleoproteins C1/C2	HNRNPC	2	7.5
45	Q12906	Interleukin enhancer-binding factor 3	ILF3	3	3.8
46	Q9UHB6	LIM domain and actin-binding protein 1	LIMA1	2	4.3
47	Q12905	Interleukin enhancer-binding factor 2	ILF2	1	4.1
48	P52907	F-actin-capping protein subunit alpha-1	CAPZA1	1	7
49	P25786	Proteasome subunit alpha type-1	PSMA1	1	4.6
50	Q8NCE2	Myotubularin-related protein 14	MTMR14	1	2.3
51	P06396	Gelsolin	GSN	1	1.4
52	P53985	Monocarboxylate transporter 1	SLC16A1	1	2.4
53	O43318-2	Isoform 1A of Mitogen-activated protein	OS	1	2.1
54	Q14103	Heterogeneous nuclear ribonucleoprotein D0	HNRNPD	2	5.4
55	P62318	Small nuclear ribonucleoprotein Sm D3	SNRPD3	1	16.7
56	Q00839	Heterogeneous nuclear ribonucleoprotein U	HNRNPU	1	1.1
57	Q13435	Splicing factor 3B subunit 2	SF3B2	1	0.8
58	Q13310	Polyadenylate-binding protein 4	PABPC4	1	2.2
59	Q96AV8	Transcription factor E2F7	E2F7	1	2.4
60	Q8N5Z5	BTB/POZ domain-containing protein KCTD17	KCTD17	1	4.7
61	Q15057	Arf-GAP with coiled-coil, ANK repeat and PH domain-containing protein 2	ACAP2	1	1.3
62	Q14681	BTB/POZ domain-containing protein KCTD2	KCTD2	1	8.4
63	P48643	T-complex protein 1 subunit epsilon	CCT5	1	2.2
64	Q9P1Y5	Calmodulin-regulated spectrin-associated protein 3	KIAA1543	1	1.7
65	Q92522	Histone H1x	H1FX	1	7.5
66	Q6IS14	Eukaryotic translation initiation factor 5A-1-like	EIF5AL1	1	7.8
67	Q13200	26S proteasome non-ATPase regulatory subunit 2	PSMD2	1	1.7
68	Q07065	Cytoskeleton-associated protein 4	CKAP4	1	2.8
69	P62805	Histone H4	HIST1H4A	1	11.7
70	P43243	Matrin-3	MATR3	1	2.2
71	O14818	Proteasome subunit alpha type-7	PSMA7	1	5.6
72	Q9Y2J2	Band 4.1-like protein 3	EPB41L3	1	0.8
73	Q9P035	Protein tyrosine phosphatase-like protein PTPLAD1	PTPLAD1	1	3
74	Q99627	COP9 signalosome complex subunit 8	COPS8	1	6.2
75	Q8N257	Histone H2B type 3-B	HIST3H2BB	1	8.7
76	Q13151	Heterogeneous nuclear ribonucleoprotein A0	HNRNPA0	1	4.9
77	P55036	26S proteasome non-ATPase regulatory subunit 4	PSMD4	1	4
78	P22392	Nucleoside diphosphate kinase B	NME2	1	7.9
79	P06748	Nucleophosmin	NPM1	1	4.4
80	O60832	H/ACA ribonucleoprotein complex subunit 4	DKC1	1	2.5
81	Q9BWJ5	Splicing factor 3B subunit 5	SF3B5	1	15.1
82	Q96I25	Splicing factor 45	RBM17	1	3.2
83	Q07021	Complement component 1 Q subcomponent-binding protein, mitochondrial	C1QBP	1	3.9
84	Q01105	Protein SET	SET	1	4.5
85	P67870	Casein kinase II subunit beta	CSNK2B	1	7.9
86	P62988	Ubiquitin	RPS27A	1	11.8
87	P60900	Proteasome subunit alpha type-6	PSMA6	1	4.9
88	P60891	Ribose-phosphate pyrophosphokinase 1	PRPS1	1	4.1
89	P54105	Methylosome subunit pICln	CLNS1A	1	5.5
90	P49721	Proteasome subunit beta type-2	PSMB2	1	6.5
91	P49458	Signal recognition particle 9 kDa protein	SRP9	1	10.5
92	P45880	Voltage-dependent anion-selective channel protein 2	VDAC2	1	6.8
93	P32969	60S ribosomal protein L9	RPL9	1	7.3
94	P31689	DnaJ homolog subfamily A member 1	DNAJA1	1	3.3
95	P28066	Proteasome subunit alpha type-5	PSMA5	1	7.9
96	P25787	Proteasome subunit alpha type-2	PSMA2	1	6
97	P23528	Cofilin-1	CFL1	1	7.2
98	P20618	Proteasome subunit beta type-1	PSMB1	1	5.8
99	P10644	cAMP-dependent protein kinase type I-alpha regulatory subunit	PRKAR1A	1	2.1
100	O95881	Thioredoxin domain-containing protein 12	TXNDC12	1	8.7
101	P25788	Proteasome subunit alpha type-3	PSMA3	1	3.9
102	O15042	U2-associated protein SR140	SR140	1	1.2
103	O76021	Ribosomal L1 domain-containing protein 1	RSL1D1	1	2.4
104	Q92841	Probable ATP-dependent RNA helicase DDX17	DDX17	1	1.7
105	P17844	Probable ATP-dependent RNA helicase DDX5	DDX5	1	1.8
106	P62258	14-3-3 protein epsilon	YWHAE	1	4.7
107	P51665	26S proteasome non-ATPase regulatory subunit 7	PSMD7	1	3.7
108	Q02543	60S ribosomal protein L18a	RPL18A	1	4.5
109	P30153	Serine/threonine-protein phosphatase 2A 65 kDa regulatory subunit A alpha isoform	PPP2R1A	1	1.4
110	P62633	Cellular nucleic acid-binding protein	CNBP	1	8.5
111	P63162	Small nuclear ribonucleoprotein-associated protein N	SNRPN	2	6.7
112	O75531	Barrier-to-autointegration factor	BANF1	1	7.9
113	P42766	60S ribosomal protein L35	RPL35	2	8.1
114	P43686	26S protease regulatory subunit 6B	PSMC4	1	3.1
115	P21333	Filamin-A	FLNA	1	0.5

116	P62847	40S ribosomal protein S24	RPS24	1	8.3
117	P51991	Heterogeneous nuclear ribonucleoprotein A3	HNRNPA3	1	3.2
118	Q9UGP8	Translocation protein SEC63 homolog	SEC63	1	1.6
119	P63173	60S ribosomal protein L38	RPL38	1	14.3

(C) Proteins associated with IRF1-FLAG

#	Uniprot ID#	Name	Gene name	Peptides (95%)	% Cov (95)
1	P10914	Interferon regulatory factor 1	IRF1	36	39.7
2	P19338	Nucleolin	NCL	8	10
3	Q13813	Spectrin alpha chain, brain	SPTAN1	6	3.4
4	P12956	X-ray repair cross-complementing protein 6	XRCC6	6	11.5
5	Q9Y2H1	Serine/threonine-protein kinase 38-like	STK38L	7	15.7
6	P51610	Host cell factor 1	HCFC1	5	2.9
7	Q01082	Spectrin beta chain, brain 1	SPTBN1	5	2.5
8	Q12906	Interleukin enhancer-binding factor 3	ILF3	4	5.4
9	Q9NR30	Nucleolar RNA helicase 2	DDX21	4	5.4
10	P09874	Poly [ADP-ribose] polymerase 1	PARP1	5	6.3
11	P16989	DNA-binding protein A	CSDA	4	9.4
12	P13010	X-ray repair cross-complementing protein 5	XRCC5	4	6.4
13	P12236	ADP/ATP translocase 3	SLC25A6	4	8.7
14	P55795	Heterogeneous nuclear ribonucleoprotein H2	HNRNPH2	3	8.7
15	P0C7M2	Putative heterogeneous nuclear ribonucleoprotein A1	HNRPA1L3	3	8.1
16	P11940	Polyadenylate-binding protein 1	PABPC1	3	3.9
17	Q06830	Peroxiredoxin-1	PRDX1	3	21.1
18	Q86V81	THO complex subunit 4	THOC4	3	15.6
19	Q00839	Heterogeneous nuclear ribonucleoprotein U	HNRNPU	3	4.5
20	O14654	Insulin receptor substrate 4	IRS4	2	2.7
21	P16402	Histone H1.3	HIST1H1D	2	9
22	Q9BTM1	Histone H2A.J	H2AFJ	2	12.4
23	P14866	Heterogeneous nuclear ribonucleoprotein L	HNRNPL	2	3.6
24	Q7Z353	Highly divergent homeobox	HDX	2	2.3
25	Q15750	TGF-beta-activated kinase 1 and MAP3K7-binding protein 1	TAB1	2	4.2
26	P62314	Small nuclear ribonucleoprotein Sm D1	SNRPD1	2	20.2
27	P22392	Nucleoside diphosphate kinase B	NME2	2	19.1
28	Q99880	Histone H2B type 1-L	HIST1H2BL	3	15.9
29	P61978	Heterogeneous nuclear ribonucleoprotein K	HNRNPK	2	5
30	P63162	Small nuclear ribonucleoprotein-associated protein N	SNRPN	2	6.3
31	O75533	Splicing factor 3B subunit 1	SF3B1	2	1.8
32	P05023	Sodium/potassium-transporting ATPase subunit alpha-1	ATP1A1	2	2.6
33	P52597	Heterogeneous nuclear ribonucleoprotein F	HNRNPF	3	8
34	P78527	DNA-dependent protein kinase catalytic subunit	PRKDC	1	0.3
35	O15294	UDP-N-acetylglucosamine--peptide N-acetylglucosaminyltransferase 110 kDa subunit	OGT	1	0.8
36	Q9NZ01	Trans-2,3-enoyl-CoA reductase	TECR	1	3.2
37	Q9NZI8	Insulin-like growth factor 2 mRNA-binding protein 1	IGF2BP1	2	3.8
38	Q9UQ35	Serine/arginine repetitive matrix protein 2	SRRM2	1	0.5
39	O15355	Protein phosphatase 1G	PPM1G	1	2.9
40	P30153	Serine/threonine-protein phosphatase 2A 65 kDa regulatory subunit A alpha isoform	PPP2R1A	1	2.2
41	Q8WWY3	U4/U6 small nuclear ribonucleoprotein Prp31	PRPF31	1	2.2
42	Q96T76	MMS19 nucleotide excision repair protein homolog	MMS19	1	0.8
43	Q08211	ATP-dependent RNA helicase A	DHX9	1	0.9
44	Q9Y520	Protein BAT2-like 2	BAT2L2	1	0.3
45	Q5JNZ5	Putative 40S ribosomal protein S26-like 1	RPS26P11	1	13
46	Q14257	Reticulocalbin-2	RCN2	1	3.5
47	P10644	cAMP-dependent protein kinase type I-alpha regulatory subunit	PRKAR1A	1	2.1
48	Q9NXV2	BTB/POZ domain-containing protein KCTD5	KCTD5	1	6.8
49	Q99615	DnaJ homolog subfamily C member 7	DNAJC7	1	2.8
50	Q92769	Histone deacetylase 2	HDAC2	1	2.5
51	Q8N5Z5	BTB/POZ domain-containing protein KCTD17	KCTD17	1	4.7
52	Q5W0B1	RING finger protein 219	RNF219	1	1.2
53	Q15369	Transcription elongation factor B polypeptide 1	TCEB1	1	9.8
54	P62805	Histone H4	HIST1H4A	1	9.7
55	P37108	Signal recognition particle 14 kDa protein	SRP14	1	9.6
56	P32969	60S ribosomal protein L9	RPL9	1	7.3
57	Q9Y3D0	Protein FAM96B	FAM96B	1	11
58	Q9ULV4	Coronin-1C	CORO1C	1	1.7
59	Q9UBX3	Mitochondrial dicarboxylate carrier	SLC25A10	1	3.5
60	Q9H8S9	Mps one binder kinase activator-like 1B	MOBKL1B	1	5.6
61	Q96HS1	Serine/threonine-protein phosphatase PGAM5, mitochondrial	PGAM5	1	4.2
62	Q8N5C8	TGF-beta-activated kinase 1 and MAP3K7-binding protein 3	TAB3	1	2.7
63	P62988	Ubiquitin	RPS27A	1	11.8
64	P62318	Small nuclear ribonucleoprotein Sm D3	SNRPD3	1	16.7
65	P62316	Small nuclear ribonucleoprotein Sm D2	SNRPD2	1	8.5
66	P62263	40S ribosomal protein S14	RPS14	1	6.6
67	P62258	14-3-3 protein epsilon	YWHAE	1	4.7

68	P53985	Monocarboxylate transporter 1	SLC16A1	1	2.4
69	P53007	Tricarboxylate transport protein, mitochondrial	SLC25A1	1	3.5
70	P49458	Signal recognition particle 9 kDa protein	SRP9	1	10.5
71	P28074	Proteasome subunit beta type-5	PSMB5	1	4.6
72	P06748	Nucleophosmin	NPM1	1	4.4
73	P05387	60S acidic ribosomal protein P2	RPLP2	1	13.9
74	P78371	T-complex protein 1 subunit beta	CCT2	1	2.8
75	P18077	60S ribosomal protein L35a	RPL35A	1	6.4
76	P62987	60S ribosomal protein L40	UBA52	1	19.2
77	P11387	DNA topoisomerase 1	TOP1	1	1.6
78	Q92522	Histone H1x	H1FX	1	7
79	P13639	Elongation factor 2	EEF2	1	1.4
80	Q93009	Ubiquitin carboxyl-terminal hydrolase 7	USP7	1	0.7
81	O95816	BAG family molecular chaperone regulator 2	BAG2	1	3.3
82	P61619	Protein transport protein Sec61 subunit alpha isoform 1	SEC61A1	1	2.1
83	Q04760	Lactoylglutathione lyase	GLO1	1	4.3
84	P25788	Proteasome subunit alpha type-3	PSMA3	1	3.9
85	Q9NQZ6	Zinc finger C4H2 domain-containing protein	ZC4H2	1	3.6
86	Q9Y3B7	39S ribosomal protein L11, mitochondrial	MRPL11	1	3.6
87	P46782	40S ribosomal protein S5	RPS5	1	3.9
88	Q9UJV8	Purine-rich element-binding protein gamma	PURG	1	2
89	Q8NC51	Plasminogen activator inhibitor 1 RNA-binding protein	SERBP1	1	2.7
90	P16615	Sarcoplasmic/endoplasmic reticulum calcium ATPase 2	ATP2A2	1	0.9
91	Q9UGP8	Translocation protein SEC63 homolog	SEC63	1	1.6
92	Q9Y221	60S ribosome subunit biogenesis protein NIP7 homolog	NIP7	1	5.6
93	P17844	Probable ATP-dependent RNA helicase DDX5	DDX5	1	1.8
94	Q71DI3	Histone H3.2	HIST2H3A	1	0
95	Q9NYF8	Bcl-2-associated transcription factor 1	BCLAF1	1	1.4
96	Q8TDN6	Ribosome biogenesis protein BRX1 homolog	BRIX1	1	2
97	P62899	60S ribosomal protein L31	RPL31	1	6.4
98	P27694	Replication protein A 70 kDa DNA-binding subunit	RPA1	1	1.9

Supplementary Table S3. siRNAs used for gene silencing.

Gene Symbol	Gene Accession	Sequence	
CSNK2B	NM_001320	CAACCAGAGUGACCUGAUU	GACAAGCUCUAGACAUGAU
		CAGCCGAGAUGCUUUAUGG	GCUCUACGGUUUCAAGAUC
TAB1	NM_153497	GAUGAGCUCUUCCGUCUUU	GAACAACUGCUUCCUGUAU
		GGAGAUUGCUGCGAUGAUU	AAAUAUGGCUACACGGACA
TAB3	NM_152787	GUACAUAGCUCAAGUGAUG	GAAAGAAGUUGACCUCCUU
		CAAAGCAACUGAAACUUGA	GAAUAGAAAUCGCCUUUUA
MAP3K7	NM_145333	CCCAAUGGCUUAUCUUACA	GGACAGCCAAGACGUAGAU
		UACACUGGAUCACCAACUA	AGUGAUAACGCGUCGGAAA
PRKDC	NM_006904	GCAAAGAGGUGGCAGUUAA	GAGCAUCACUUGCCUUUAA
		GAUGAGAAGUCCUUAGGUA	GCAGGACCGUGCAAGGUUA
XRCC5	NM_021141	GGAAGAAGCCAUUAAGUUU	GAAGUGAUAUAGUUCCUUU
		GAUGAUAUCUUUAGAAGGU	GCUCAUAAAUCACAUCGAA
XRCC6	NM_001469	GAAGGAGGUUGCAGCAUUG	GGCCCAAGGUGGAGUAUUC
		GAAGAUGAGUUGACACCUU	ACAGAGAUAUCAUCAGCAU
PARP1	NM_001618	GAAAGUGUGUUCAACUAAU	GCAACAAACUGGAACAGAU
		GAAGUCAUCGAUAUCUUUA	GAUAGAGCGUGAAGGCGAA
NME2	NM_001018139	GAAGACAGGCCGAGUGAUG	GCUCAUGACUGGGUCUAUG
		GCGAGAUCAUCAAGCGCUU	GAAAUCAGCCUAUGGUUUA
SPTAN1	NM_003127	GCAAAGAUCUUACCAAUGU	CAACAGAGGUAAGGAUUUA
		GAGAGGAACUGAUUACAAA	UGACAACACCAUCGGGAAA
SPTBN1	NM_178313	CGGAAGAGAUCGCCAAUUA	GACGAGAUCUUGUGGGUUG
		CUUAUGUGGUGACUUAUUA	CGAGUGCAAUGAAACCAAA
BCLAF1	NM_001077441	GAACAUAGUACUCGGCAAA	GGAAUGAGACGACCUUAUG
		UAGUAGAGAUCGUAUGUAU	GUAAAUUGUCAUACGCCAA
STK38L	NM_015000	GAAAGAAACUCUGGUAUUU	GAAACACAGUUCUACAUUU
		GCAGACUGGUUACAACAAA	AAAGUUACGUCGAUCACAA
YWHAE	NM_006761	GCUUAGGUCUUGCUCUCAA	GUGAUAUUGCAAUGACAGA
		GCUGAGUGAAGAAAGCUAU	UAGCAGGGAUGGAUGUGGA
PRKAR1A	NM_002734	CGAGACAGCUAUAGAAGAA	GAUAAUGAGAGAAGUGAUA
		UACGGUAGCUGAUGCAUUG	GUGGGAACGUCUUACGGUA
HCFC1	NM_005334	GAACAACAUUCCAAGGUAC	CGAAGUAGACCAGUUAUCA
		CCGGCAAGAUUAUCGAGUA	CGAAAUCUCAGCCUUUAAG
NCL	NM_005381	CAAAUCUGCUCCUGAAUUA	GAAAUAAAGUUACCUUGGA
		GAAAGAAGACGAAGUUUGA	GGAAGAGCCUGUCAAAGAA
DDX21	NM_004728	GGAAGAAUAUCAGUUAGUA	GGAAUUAAGUUCAAACGAA
		GGAGUGACCUUCCUAUUUC	CAAACUAGAUCUCACCAAA
PRDX1	NM_002574	GAACAUUCCUUUGGUAUCA	AAACUCAACUGCCAAGUGA
		GUAAAUGACCUCCCUGUUG	GGAGAUCAUUGCUUUCAGU

ILF3	NM_153464	GACCGAAAUUUGCUGCUAA	GGAGGUUGAUGGCAAUUCA
		AGACAGACAGCAACGGGAA	GAGCAGGCAGAGUCCGAUA
CSNK2A1	NM_001895	GAUCCACGUUUCAAUGAUA	
CSNK2A2	NM_001896	CCUAGAUCUUCUGGACAAA	
CSNK1A	NM_001025105	GGCUAAAGGCUGCAACAAA	
CSNK1E	NM_001894	GCGACUACAACGUGAUGGU	
CSNK1G1	NM_022048	GAGAUGAUUUGGAAGCCCU	
CSNK1G2	NM_001319	GCGAGAACUUCCCAGAGGA	
CSNK1G3	NM_001031812	CUUACAGGAACAGCUAGAU	
Csnk2b	NM_009975.3	CUCUGGACAUGAUCUUAGA	AGAGCGACUUGAUCGAACA
		GGACAAAUUUAAUCUUACU	
AFAP1	NM_001134647	GGCUGUAACAUUACGUACA	GGACGGCACUUCAUUAUGA
PLAAT4	NM_004585	UAUGGCAAGUCCCGCUGUA	
IRF1	NM_002198	UCACAGAUCUGAAGAACAU	CCAAGAACCAGAGAAAAGA
Non-targeting Control	N/A	UAGCGACUAAACACAUCAA	AUGUAUUGGCCUGUAUUAG
		UAAGGCUAUGAAGAGAUAC	AUGAACGUGAAUUGCUCAA

Supplementary Table S4. Single guide RNAs used for CRISPR/Cas9 knockouts.

Target gene	Target sequence
Non-target control	CACTCACATCGCTACATGA
CSNK2B (#1)	TCTAGAGCTTGTCGATAGTG
CSNK2B (#2)	TCACCATCTGGGCGATGCCA
CSNK2B (#3)	CGCTACATCCTTACCAACCG
CSNK2B (#4)	GGGGCAGTAGAGCTTCACCA

Supplementary Table S5. Oligonucleotide primers used for qRT-PCR of host genes.

Gene	Forward	Reverse
PLAAT4	GATTTTCCGCCTTGGCTATG	TTGCTCAGGACTGAGAAGAC
PSMB9	GTGGATGCAGCATATAAGCC	AGTGACCAGGTAGATGACAC
AFAP1	TCAACTCGCAGCTCAAGGGT	GCATCTGCTTCTACCCGGTTCT
GBP3	CGCACAGGAAAATCCTACCT	ACACACCACATCCAGATTCC
ERAP2	GGGCCTCATTACATATAGGGA	ATTCCATTGTGACCAGGTTG
AHR	AGAGGTGCTTCATATGTCGT	AGTGGAGTAGCTATCGCAAA
DNAJA1	TACATCAGCTCTCAGTAACC	TCTACTGCTCCTTTCTTACC
DNPEP	GGACAAGCATGAGGAGAACC	ACACCGCGTTTGAAGCATAG
ELF1	GTCATGGAGGATGAACGACA	CTCTTCCACACAGGCTAGAC
HMGB1	TGCAGTACATTGAGCTCCATAG	TCTTATGCTCCTCCCGACAA
PARP14	TTTCCTTGACAGCCTAGCGT	ACCTGGCACACAATGTAGGT
SECTM1	GCTGTCTTCATCCTCTTGGT	CCTTCATCTGGGGTTCTAGG
SRP54	AGAATGTGAGCCAGTCACAG	CCTGTTGAAACTGCCTCATC
TAP2	CCTCACTATTCTGGTCGTGT	GATCCGCAAGTTGATTCGAG
SYNCRIP	AACAAGGGACCAAAGTAGCA	CTCTGTCCAGTGGTCACATC
TFAP2A	CGTTACCCTGCTCACATCAC	TGCTTTGGCAGGAAATTCGG
MX1	CAGTTACCAGGACTACGAGA	GGGTGATTAGCTCATGACTG
ZNFX1	AGAAATCAGGCCAATAACCC	TCTTTGGTCTCTAGCTTCGT
LGALS3BP	ACCATGAGTGTGGATGCTGA	TGGAAGCACTTGACTGACGA
DDX60L	GGGCATGAATCTGATACTCTC	GATCCTTCTGACCATAGGTG
NLRC5	CCAAGTTCTTAGGGTTCCGT	CTCTGGTCACTCAGGAACTC
IFI6	GGTCTGCGATCCTGAATGGG	TCACTATCGAGATACTTGTGGGT
STAT1	CCATCCTTTGGTACAACATGC	TGCACATGGTGGAGTCAGG
PDL1	GCACTGACATTCATCTTCCGTT	AGTTCCAATGCTGGATTACGTC
CSNK2B	AGGTCCCTCACTATCGACAA	CCTGCTCAATCAGGTCACTC
XRCC6	ATCTCTTGGCTGTGGTGTTC	CTGCCCCTTAAACTGGTCAA
TAB1	AGGTGACACAGCTGAACGTG	GATGATCCCCACCTGCTTGA
TAB3	GAGAAAAGCCCGAAGAATTAGCGT	ATTCCATGGAGCCCCTTCGT
MAP3K7	GGAGCAAGTTCCTGCCACAA	AGGCTCCCAAGCTTAAACGTC
XRCC5	CACAGACATCTGATGCTACCA	AATCACATCCATGCTCACGA
STK38L	ACCAGTGCCAAATACCACAGA	TAGAGCCACGTTGAGTCAACC
PRKAR1	ATCCTCATGGGAAGCACACT	TGCACTGGTTCCAATGCATC
DDX21	TTGGAACACCAGGTCGTATC	TCCACTTCATCCAGGACAAC
HCFC1	TGTGCAGCCTATGGCTTCGT	GTTGTTCTTTGGGTCCTCGCTATC
